# *Leishmania major* and *Trypanosoma lewisi* infection in invasive and native rodents in Senegal

**DOI:** 10.1371/journal.pntd.0006615

**Published:** 2018-06-29

**Authors:** Cécile Cassan, Christophe A. Diagne, Caroline Tatard, Philippe Gauthier, Ambroise Dalecky, Khalilou Bâ, Mamadou Kane, Youssoupha Niang, Mamoudou Diallo, Aliou Sow, Carine Brouat, Anne-Laure Bañuls

**Affiliations:** 1 MIVEGEC, IRD, CNRS, Univ. Montpellier, Montpellier, France; 2 CBGP, IRD, CIRAD, INRA, Montpellier SupAgro, Univ Montpellier, Montpellier, France; 3 CBGP, IRD, CIRAD, INRA, Montpellier SupAgro, Univ Montpellier, Campus ISRA/IRD de Bel Air, Dakar, Sénégal; 4 Département de Biologie Animale, Faculté des Sciences et Techniques, Université Cheikh Anta Diop (UCAD), Dakar, Sénégal; 5 CBGP, INRA, CIRAD, IRD, Montpellier SupAgro, Univ Montpellier, Montpellier, France; 6 IRD, LPED (UMR AMU/IRD), Marseille, France; Universiteit Antwerpen, BELGIUM

## Abstract

Bioinvasion is a major public health issue because it can lead to the introduction of pathogens in new areas and favours the emergence of zoonotic diseases. Rodents are prominent invasive species, and act as reservoirs in many zoonotic infectious diseases. The aim of this study was to determine the link between the distribution and spread of two parasite taxa (*Leishmania* spp. and *Trypanosoma lewisi*) and the progressive invasion of Senegal by two commensal rodent species (the house mouse *Mus musculus domesticus* and the black rat *Rattus rattus*). *M*. *m*. *domesticus* and *R*. *rattus* have invaded the northern part and the central/southern part of the country, respectively. Native and invasive rodents were caught in villages and cities along the invasion gradients of both invaders, from coastal localities towards the interior of the land. Molecular diagnosis of the two trypanosomatid infections was performed using spleen specimens. In the north, neither *M*. *m*. *domesticus* nor the native species were carriers of these parasites. Conversely, in the south, 17.5% of *R*. *rattus* were infected by *L*. *major* and 27.8% by *T*. *lewisi*, while very few commensal native rodents were carriers. Prevalence pattern along invasion gradients, together with the knowledge on the geographical distribution of the parasites, suggested that the presence of the two parasites in *R*. *rattus* in Senegal is of different origins. Indeed, the invader *R*. *rattus* could have been locally infected by the native parasite *L*. *major*. Conversely, it could have introduced the exotic parasite *T*. *lewisi* in Senegal, the latter appearing to be poorly transmitted to native rodents. Altogether, these data show that *R*. *rattus* is a carrier of both parasites and could be responsible for the emergence of new foci of cutaneous leishmaniasis, or for the transmission of atypical human trypanosomiasis in Senegal.

## Author summary

Biological invasions (the introduction and adaptation of living organisms to a new environment) are increasingly frequent due to worldwide intensification of human-associated exchanges. They can lead to the introduction of pathogens in new areas and favour the emergence of diseases. Rodents are prominent invasive species, and act as reservoirs in many infectious diseases. The aim of our study was to determine the link between the distribution and spread of two parasites, *Leishmania* spp. and *Trypanosoma lewisi*, and the progressive invasion of Senegal by two commensal rodent species, the house mouse *Mus musculus domesticus*, and the black rat *Rattus rattus*. We identified *R*. *rattus* as a potential reservoir for *Leishmania major* and *T*. *lewisi* in the southern part of Senegal. The presence of these two pathogens in *R*. *rattus* may be of different origins. The invader *R*. *rattus* could have been locally contaminated with *L*. *major*. Conversely, *T*. *lewisi* infection could have been introduced in Senegal by *R*. *rattus*, and seems to be poorly transmitted to native rodents. Altogether, these data show that *R*. *rattus* is a carrier of both parasites, and could be responsible for the emergence of new foci of cutaneous leishmaniasis or for the transmission of atypical human trypanosomiasis in Senegal.

## Introduction

Biological invasions are increasingly frequent due to the worldwide intensification of human-associated exchanges. They can have significant consequences on the biodiversity in many ecosystems [1]. For instance, they might lead to novel parasite-host combinations and have dramatic effects on the dynamics of diseases that affect wildlife, livestock and/or humans [2]. Disease emergence events associated with exotic pathogens imported by animal invaders have already been reported [3]. The dynamics of endemic diseases could also be affected by invasive species that act as novel hosts, or could negatively affect native host species [4].

Among invasive species, rodents indisputably represent the vertebrate group that has most often accompanied the global dispersion of humankind. Rodents act as reservoir for numerous zoonotic agents, such as helminths, bacteria or protozoa. The introduction of exotic rodents has been associated with the appearance of several new foci of infectious diseases in wildlife (e.g., [5,6,7]) and human populations (e.g., [8]). However, up to now, only few studies have evaluated the effects of both native and invasive host communities on infectious disease risk in invaded ecosystems. For instance, Billeter et al. [9] showed that the risk of transmission of bartonellosis to human was higher from native rodents than from invasive black rat in Uganda. In this study, we focused on the spatial distribution of two zoonotic protozoan parasites, *Leishmania* spp. and *Trypanosoma lewisi*, in native and invasive populations of commensal rodents in Senegal.

Leishmaniasis is a neglected disease that affects 0.9 to 1.6 million people worldwide and responsible for 20,000 to 40,000 deaths each year [10]. Moreover, 350 million people are considered at risk of contracting this infection [11]. Among the 30 *Leishmania* species described in mammals, 20 are pathogenic for humans [12,13]. These species are generally host-specific and restricted to particular geographical areas. The parasite is transmitted through the bite of infected female phlebotomine sand flies. In humans, they can cause different clinical forms: asymptomatic infection, visceral leishmaniasis (VL), mucocutaneous leishmaniasis (MCL) or cutaneous leishmaniasis (CL) [13]. VL is mainly caused by *Leishmania infantum* and *Leishmania donovani*. Human VL has never been detected in Senegal [10,14,15], but high human serological prevalence of *Leishmania* has been found in a focus near Thiès [16], where canine leishmaniasis caused by *L*. *infantum* has been recorded since last century and recently epidemiologically described [14,16,17,18]. Atypical MCL due to *Leishmania major* (the main causative agent of MCL is *Leishmania braziliensis*, whose distribution is restricted to South America [13]) can occur in Senegal but is very rare (Dr. Babacar Faye, personal communication, and [19]). Rural CL is caused by *Leishmania major*, and is endemic in West and North Africa, and in the Middle East. In Senegal the infection has been observed since the beginning of the 20^th^ century [20,21,22,23,24] and is still reported [25, Dr Babacar Faye, personal communication]. Nevertheless, public health records about the prevalence and distribution of the disease remain limited.The epidemics appear as foci that then disappear, possibly in function of seasons, and dynamics of vectors and reservoir populations [21,24]. Gerbils are traditionally considered to be the *L*. *major* reservoirs in the Old World [11]. However, the parasite was identified in various rodent species (e.g., *Mastomys erythroleucus*, *Arvicanthis niloticus* and *Gerbilliscus gambianus*) near Thiès in Senegal [21,26,27]. These asymptomatic rodents (showing no cutaneous lesion) were found infected, by culture or molecular methods. In Senegal, *L*. *major* is transmitted by the vector *Phlebotomus duboscqi* [28]. *P*. *duboscqi* is found mainly in rodent burrows, termite mounds and tree holes, but also indoor; it feeds on many vertebrates, birds, reptiles and mammals (rabbits, rodents) and is also very anthropophilic. [18,28,29].

The genus *Trypanosoma* includes pathogens, such as *Trypanosoma gambiense*, *T*. *cruzi* and *T*. *brucei brucei*, responsible for sleeping sickness, Chagas’ disease and African animal trypanosomiasis, or nagana in livestock respectively, as well as other species considered to be non-pathogenic for humans [30]. However, atypical human trypanosomiases that are caused by species normally restricted to animals, for instance *T*. *lewisi*, have been recently described [31]. Humans are rare accidental hosts for *T*. *lewisi*, and only a few cases have been reported worldwide [30]. The infection is generally associated with mild symptoms, such as fever for a few days, but can require medical intervention in young children, and sometimes can lead to death [30]. *T*. *lewisi* has been identified for the first time in France in the 19^th^ century, then in Poland in 1880 and 1901 [32]. It is now found in nearly all continents, including Africa where it was detected in rodents in Niger [33] and Nigeria [34,35], and in one infant in Gambia [36]. Nothing more is known on its presence in West Africa, and no human infection has been reported in Senegal up to now [30], but this atypical trypanosomiasis could be under-diagnosed, possibly due to asymptomatic carriage or to non specific symptoms. *T*. *lewisi* is described as quite host-specific, and infects rodents, mainly rats [37]. In rodents, its pathogenic potential is low. This parasite proliferates in blood and is rarely found in organs. *T*. *lewisi* is orally transmitted to rodents by ingestion of hematophagous arthropods or their faeces [38].

In Senegal, villages and towns are invaded by two major invasive rodent species (Global Invasive Species Database [39]): the house mouse *Mus musculus domesticus* and the black rat *Rattus rattus*. Historical records and molecular analyses have shown that these rodents were first brought to sea ports in Senegal by Europeans during the colonial period [40,41,42]. Over the last century, both taxa have spread to inland villages and towns due to the improvement of transport infrastructures, and the native commensal rodent species have been progressively evicted from the invaded habitats [41].

The goal of this study was to evaluate *Leishmania* and *T*. *lewisi* infection rates in commensal native and invasive rodent communities of Senegal in localities along invasion gradients, in order (i) to explore the link between rodent invasion and the spread of these two trypanosomatids, and (ii) to assess the health risk for human populations.

## Materials and methods

### Ethical statement

Trapping campaigns within localities and private land were conducted with the authorization of the appropriate institutional and household authorities. They were carried out under the framework agreement established between the Institut de Recherche pour le Développement and the Republic of Senegal, as well as with the Senegalese Head Office of Waters and Forests. None of the rodent species investigated here has protected status (see list of the International Union for Conservation of Nature). Handling procedures were performed under our lab agreement for experiments on wild animals (no. 34-169-1), and followed the official guidelines from the American Society of Mammalogists [43]. Euthanasia was performed as recommended by the Federation of European Laboratory Animal Science Associations (FELASA) for small rodents [44,45]. Sample transfers have been approved by the regional Head of Veterinary Service (Hérault, France).

### Distribution of the rodents and sampling

**Fig 1:**
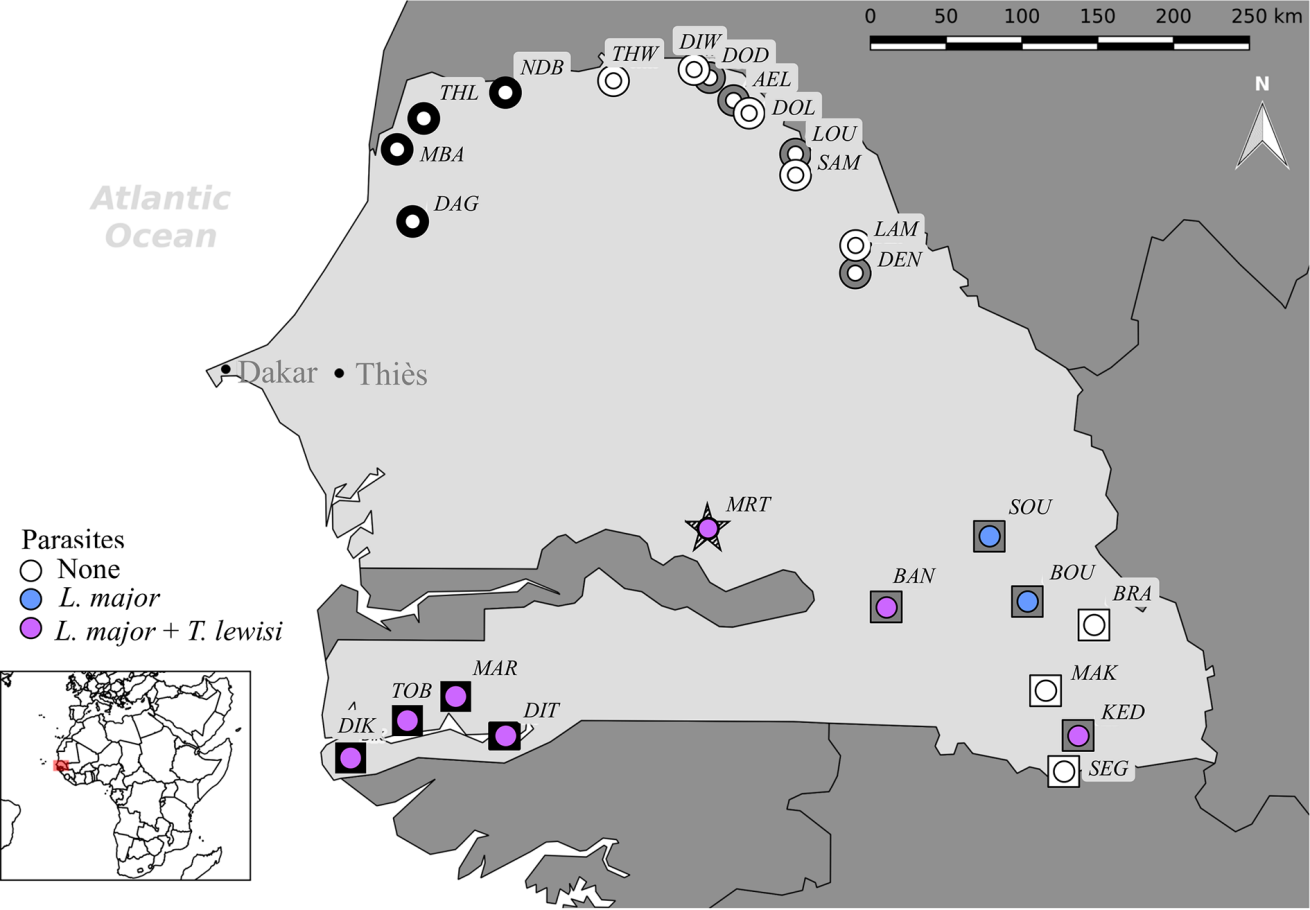
Rodent sampling localities along the house mouse (*Mus musculus domesticus*) (circles) and black rat (*Rattus rattus*) (squares) invasion gradients in Senegal (West Africa). Colour code: black for localities of long-established invasion (LI: rodent communities dominated by invasive species); grey for recently invaded localities or the invasion front (IF: sympatric areas with invasive rodents and native *Mastomys erythroleucus*, or *Mastomys natalensis* in KED); white for non-invaded localities (NI: only native rodents, *Ma*. *erythroleucus* in the north, *Ma*. *natalensis* in the south). Presence (blue dots for *Leishmania major*; violet dots for *Trypanosoma lewisi* + *L*. *major*) and absence (white dots) of parasites in the rodent species are also indicated. AEL = Aere Lao, BAN = Badi Nieriko, BOU = Boutougoufara, BRA = Bransan, DAG = Dagathie, DEN = Dendoudi, DIK = Diakène Wolof, DIT = Diattacounda, DIW = Diomandiou Walo, DOD = Dodel, DOL = Doumga Lao, GAL = Galoya, KED = Kedougou, LAM = Lambango, LOU = Lougue, MAK = Mako, MAR = Marsassoum, MBA = Mbakhana, NDB = Ndombo, SAM = Sare Maoundé, SEG = Segou, SOU = Soutouta, THL = Thilene, THW = Thiewlé, TOB = Tobor. MRT (= Mereto; hatched star) is invaded by both *M*. *m*. *domesticus* and *R*. *rattus*. The map was created using the QGIS software v. 2.18.7 (http://www.qgis.org/fr/site/).

The house mouse is now present in most of northern and central Senegal, whereas the black rat is distributed throughout the southern part of the country (**Fig 1**). Rodents were live-trapped in localities (villages and towns) along each of these two invasion gradients. We used data from historical records and longitudinal sampling surveys of rodent communities in Senegal carried out since the 1980s [41,46,47], in order to classify sampling localities into three categories related to invasion status: (i) in long-established invasion localities (LI), the house mouse or the black rat settled in large and permanent populations were present since more than a century, and have excluded native rodents; (ii) in recently invaded localities corresponding to invasion front (IF), exotic rodents arrived only recently (10–30 years ago) and currently coexist with native rodents; (iii) in non-invaded localities (NI), the house mouse and the black rat have never been detected, and only native rodents are known to occur. Three to six localities were systematically sampled per invasion category along each invasion gradient (**Fig 1**). In addition, rodents were trapped also in Mereto, a village in the Terres Neuves region (star in **Fig 1**). The lack of data on rodent communities in this village (it was not sampled for rodents before this study) prevented its classification into a specific invasion category: the village was created before 1972 [48], so exotic rodents may have arrived there for more than 40 years, or later.

Sampling was performed between March and April 2013 in the north, and between November 2013 and February 2014 in the south (including Mereto). Details on rodent trapping and identification, autopsy procedures and age determination are provided elsewhere [41,46,49]. Young animals were eliminated from the study. Each rodent was euthanized by cervical dislocation and dissected.

### Sample preparation

For this study, the spleen and one ear from adult animals were collected. Tissues were stored at 4°C in 95% ethanol prior to use. DNA extraction was performed with the DNeasy Blood and Tissue Kit according to the manufacturer’s protocol (Qiagen, Courtaboeuf, France).

To verify the DNA quality and validate negative results, we performed a real time PCR targeting the rodent β-actin gene in all samples collected in southern Senegal with the primers bAqF (5’-TCCGTAAAGACCTCTATGCCAA-3’) and bAqR (5’-CAGAGTACTTGCGCTCAG-3’) [50] on a 7300 Real-Time PCR instrument (Applied Biosystems, Foster City, USA). Each 8μl reaction mix included 1.6μl of 5X Evagreen (Euromedex, Souffelweyersheim, France), 0.375μM of each primer, and 2μl of 100 times diluted DNA (about 5-10ng). Cycling conditions were: 15min initial denaturation followed by 45 amplification cycles (95°C for 10s, 60°C for 15s, 72°C for 30s) and a melting curve (95°C for 15s, 60°C for 30s, 5 acquisitions per sec. up to 95°C at 0.11°C/sec). Fluorescence detection was performed at the end of the amplification step. The expected size of the amplicon was 274b. Actin could not be amplified in 20 samples that were thus re-extracted to improve quality.

### Molecular diagnosis of *Leishmania*

*Leishmania* detection was performed in spleen and a few ear samples. For *Leishmania* diagnosis, a highly sensitive nested PCR method to amplify the minicircle kinetoplastic DNA (kDNA) variable region was chosen, as described by Noyes et al. [51]. For the first PCR step, the primers CSB1XR (5’-ATT TTT CSG WTT YGC AGA ACG-3’) and CSB2XF (5’-SRT RCA GAA AYC CCG TTC A-3’) were used and the following conditions: 2min initial denaturation at 94°C, followed by 45 amplification cycles (94°C for 30s, 54°C for 1min, 72°C for 1min) and a final extension step of 72°C for 10min. Each 30μl reaction mix included 0.333μM of each primer, 42μM of each dNTP, 3μl of 10X buffer, 1U of Taq DNA polymerase (Roche Diagnostics, Meylan, France), and 3μl of template DNA. For the second PCR step, 3μl of the first PCR product were used with the same programme (but for the annealing temperature that was increased to 56°C) and the same reaction mix except the primers that were replaced by the following: LIR (5’-TCG CAG AAC GCC CCT-3’) and 13Z (5’-ACT GGG GGT TGG TGT AAA ATA G-3’). For this second step, the expected size of the amplicon was 500-800b depending on the *Leishmania* species. *Leishmania* complex identification was based on comparison of the size of the second PCR product on a 1.5% agarose gel in 0.5X TAE buffer with the reference strain profiles [51]. To confirm the diagnosis, amplicons from positive samples were cloned in the pGEM-T vector (Promega, Charbonnières-les-Bains, France) and sequenced with the LIR and 13Z primers. BLAST was used to compare the obtained sequences to those included in the NCBI and TriTrypDB databases. BLAST results were taken into account when they came from reference strains or from sequences published in peer-reviewed articles, and were well characterized in terms of species, origin and hosts, in order to avoid misidentifications. Our sequence data were also compared with sequences obtained with the same method from two *L*. *major* reference strains (MHOM/SU/73/5ASKH and MHOM/IL/1980/Friedlin) and from a *L*. *major* strain isolated from a Senegalese patient (LC-DKR). This last strain was isolated in 2008 at the Dermatology unit of the hospital A. Le Dantec in Dakar by Pr Babacar Faye (laboratory of Parasitology and Mycology, University Cheikh Anta Diop, Dakar) from a patient with typical *L*. *major* lesions; *L*. *major* species was confirmed with the nested PCR described above). The experimental clones were named using the field sample number to which a suffix was added. Sequences were submitted to GenBank (**S1 Table, S1 Text**).

### Molecular diagnosis of *Trypanosoma lewisi*

*T*. *lewisi* detection was performed in spleen. Diagnosis in mouse samples was performed using a FRET-based real time PCR method to detect 18S rRNA with the primers TRYP A1 (5'-AGGAATGAAGGAGGGTAGTTCG-3’) and TRYP A2 (5'-CACACTTTGGTTCTTGATTGAGG-3') and the probes TRYP A3 (5’-*LC640*AGAATTTCACCTCTGACGCCCCAGT*Phos-3’*) and TRYP A4 (5’-GCTGTAGTTCGTCTTGGTGCGGTCT*Flc-3’*) [33], on a LightCycler LC 480 instrument (Roche Diagnostics, Meylan, France). Each 12μl reaction mix included 6μl of 2X Maxima Probes master mix (Thermo Fisher Scientific, Waltham, Massachusetts, USA), 0.5μM of each primer, 0.25μM of each probe, and 5μl of template DNA. After enzyme activation at 50°C for 1min and an initial denaturation step of 95°C for 10min, 50 amplification cycles were carried out at 95°C for 10s, 56°C for 10s, 72°C for 15s, followed by a melting curve (95°C for 15s, 56°C for 30s, 5 acquisitions per sec. up to 95°C at 0.11°C/sec). Fluorescence acquisition was performed with a Red 640 filter at the end of the annealing step. The expected size of the amplicon was 131bp.

The FRET-based real time PCR was chosen for its robustness and sensitivity (<9.5fg, **S1 Fig**). However, this PCR is not specific for *T*. *lewisi*, but detects also *Leishmania* (**S1 Fig**). This latter parasite was not detected in samples from the first campaign realized in the north of Senegal, but was detected in some of those from the second campaign in the central/south of Senegal (see Results). Therefore, we developed a more specific approach based on real time PCR amplification of *T*. *lewisi* mini-exon instead of the FRET-based real time PCR for this second campaign. The mini-exon amplification was realized with the primers ME-F (5'-GCTGACACCGGTTGGTTCTG -3’) and ME-R (5'-GACAGCAGCGTGAGCAATAA -3'). The 8μl reaction mix included 4μl 2X SYBR Green (Roche Diagnostics, Meylan, France), 0.6μM of each primer, and 2μl of template DNA and the following cycling conditions: initial denaturation step of 95°C for 5min, followed by 50 amplification cycles (95°C for 15s, 59°C for 20s, 72°C for 15s) and a melting curve (95°C for 5s, 59°C for 1min, 5 acquisitions per sec. up to 95°C at 0.11°C/sec) on a LightCycler LC 480 instrument (Roche Diagnostics, Meylan, France). Fluorescence acquisition was performed at the end of the amplification step. This PCR was specific to *T*. *lewisi* with a sensitivity of 1.9fg. The expected size of the amplicon was 140bp. Results were confirmed by amplicon sequencing (**S2 Text**) and comparison with sequence data from the NCBI database; sequences obtained showed homologies with the mini-exon sequence of the strain Molteno B3 (Genbank AJ250740.1).

### Statistical analysis

Statistical analyses were restricted to *R*. *rattus* as positive samples for both *Leishmania* and *T*. *lewisi* were mainly identified in this host species (see Results). Generalized linear mixed models (GLMMs) assuming a binomial distribution were used to test whether prevalence levels (infected/non-infected) differed among invasion categories. The sampling locality was considered as a random effect. *P*-values were obtained by stepwise model simplification in likelihood-ratio tests and were considered significant when <0.05. The models were validated by checking the normality, independence and variance homogeneity of the residuals. All model analyses were performed with the R software v 3.2.2 [52] using the package lme4 v1.1–14 [53].

## Results

During the two sampling campaigns, the following commensal native rodents were trapped: *Ma*. *erythroleucus* in northern Senegal, and *Ma*. *erythroleucus*, *Mastomys natalensis*, *A*. *niloticus* and *Praomys daltoni* in southern or central Senegal (see Dalecky et al. 2015 [41] for a detailed description of commensal rodent communities in Senegal). Molecular diagnosis was performed on tissue specimens from 161 *M*. *m*. *domesticus* and 167 *Ma*. *erythroleucus* samples in the north (**Table 1**), and from 241 invasive rodent (212 *R*. *rattus*, 29 *M*. *m*. *domesticus*) and 219 native rodent (96 *Ma*. *natalensis*, 67 *Ma*. *erythroleucus*, 12 *A*. *niloticus* and 15 *P*. *daltoni*) samples in the south (**Table 1**) (**Fig 1**). Mereto was the only locality where native rodents coexisted with both mice and rats.

**Table 1:** Summary of rodents trapped in localities of *Mus musculus domesticus* or *Rattus rattus* long-established invasion (LI), invasion front (IF) and no invasion (NI).

Northern Senegal						Southern Senegal							
Locality category	Locality	*Mastomys erythroleucus*			*Mus musculus domesticus*	Total	Locality	*Arvicanthis niloticus*	*Mastomys erythroleucus*	*Mastomys natalensis*	*Mus musculus*	*Praomys daltoni*	*Rattus rattus*	Total
	Dagathie	-			20	20	Diakene Wolof	-	3	-	-	1	24	28
*LI*	Mbakhana	-			22	22	Diattacounda	-	13	-	-	1	27	41
	Ndombo	-			20	20	Marsassoum	-	1	-	-	1	26	28
	Thilene	-			20	20	Tobor	-	-	-	-	2	21	23
	***Total LI***	***-***			***82***	**82**	***Total LI***	***-***	***17***	***-***	***-***	***5***	***98***	**120**
	Aere Lao	20			20	40	Badi Nieriko	-	13	-	-	-	24	37
*IF*	Dendoudi	22			17	39	Boutougoufara	-	19	23	-	5	31	78
	Dodel	20			20	40	Kedougou	-	-	-	-	-	23	23
	Lougué	21			22	43	Soutouta	6	11	-	-	1	23	41
	***Total IF***	***83***			***79***	**162**	***Total IF***	***6***	***43***	***23***	***-***	***6***	***101***	**179**
	Diomandou Walo	14			-	14	Bransan	-	3	23	-	1	-	27
*NI*	Doumga Lao	20			-	20	Mako	-	-	26	-	-	-	26
* *	Lambango	20			-	20								
* *	Saré Maoundé	9			-	9								
* *	Thiewle	21			-	21								
*** ***	***Total NI***	***84***			***-***	**84**	***Total NI***	***-***	***3***	***73***	***-***	***4***	***-***	**80**
							Mereto	6	4	-	29	-	13	**52**
	**Total**		**167**	**161**		**328**	**Total**	**12**	**67**	**96**	**29**	**15**	**212**	**431**

### *Leishmania* diagnosis

None of the trapped rodents presented any clinical sign of leishmaniasis. Molecular diagnosis was first performed on spleen samples. All rodents sampled in the north of Senegal (*Ma*. *erythroleucus* and *M*. *m*. *domesticus*) were negative for *Leishmania* (**Fig 1**).

Conversely, in the south, 37 *R*. *rattus*, one *M*. *m*. *domesticus* (from Mereto) and two native rodent samples (*Ma*. *erythroleucus* in Soutouta; *A*. *niloticus* in Mereto) were positive for *Leishmania* (**Table 2** and **Fig 1**). The positive samples represented 12.5% and 11.2% of all specimens in the LI and IF localities, respectively, excluding Mereto. No *Leishmania* infection was detected in samples from NI localities (**Fig 1**). In *R*. *rattus* samples (the main host), *Leishmania* prevalence was similar between LI and IF localities (*F*_1.199_ = 0.43, *p =* 0.511).

**Table 2:** *Leishmania major* and *Trypanosoma lewisi* infection in rodents trapped in southern Senegal. Number of positive/total number of rodents in localities of *Rattus rattus* long-established invasion (LI), invasion front (FI) and no invasion (NI), and in Mereto.

Locality category	Locality	*Leishmania major*							*Trypanosoma lewisi*										Double infected *Rattus rattus*
		*Arvicanthis niloticus*	*Mastomys erythroleucus*	*Mastomys natalensis*	*Mus musculus*	*Praomys daltoni*	*Rattus rattus*	Total	*Arvicanthis niloticus*	*Mastomys erythroleucus*	*Mastomys natalensis*	*Mus musculus*	*Praomys daltoni*	*Rattus rattus*		Total			
	Diakene Wolof	-	0/3 (0%)	-	-	0/1 (0%)	5/24 (20.8%)	5/28 (17.9%)	-	0/3 (0%)	-	-	0/1 (0%)	14/24 (58.3%)		14/28–50%)			4/24 (16.7%)
***LI ***	Diattacounda	-	0/13 (0%)	-	-	0/1 (0%)	6/27 (22.2%)	6/41 (14.6%)	-	0/13 (0%)	-	-	0/1 (0%)	8/27 (29.6%)		8/41 (19.5%)			2/27 (7.4%)
*** ***	Marsassoum	-	0/1 (0%)	-	-	0/1 (0%)	1/26 (3.8%)	2/28 (7.1%)	-	0/1 (0%)	-	-	0/1 (0%)	6/26 (23.1%)		6/28 (21.4%)			1/26 (3.8%)
*** ***	Tobor	-	-	-	-	0/2 (0%)	3/21 (14.3%)	3/23 (13.0%)	-	-	-	-	0/2 (0%)	6/21 (28.6%)		6/23 (26.1%)			2/21 (9.5%)
*** ***	***Total locality LI***	***-***	***0/17 (0%)***	***-***	***-***	***0/5 (0%)***	***15/98 (15*.*3%)***	**15/120 (12.5%)**	***-***	***0/17 (0%)***	***-***	***-***	***0/5 (0%)***	***34/98 (34*.*7%)***		**34/120 (28.3%)**			**9/98 (9.2%)**
	Badi Nieriko	-	0/13 (0%)	-	-	-	4/24 (16.7%)	4/37 (10.8%)	-	0/13 (0%)	-	-	-	15/24 (62.5%)		15/37 (40.5%)			2/24 (8.3%)
***IF ***	Boutougoufara	-	0/19 (0%)	0/23 (0%)	-	0/5 (0%)	7/31 (22.6%)	7/78 (8.9%)	-	0/19 (0%)	0/23 (0%)	-	0/5 (0%)	0/31 (0%)		0/78 (0%)			0/31 (0%)
*** ***	Kedougou	-	-	-	-	-	5/23 (21.7%)	5/23 (21.7%)	-	-	-	-	-	10/23 (43.5%)		10/23 (43.5%)			1/23 (4.3%)
*** ***	Soutouta	0/6 (0%)	1/11 (9.1%)	-	-	0/1 (0%)	3/23 (13.0%)	4/41 (9.8%)	0/6 (0%)	0/11 (0%)	-	-	0/1 (0%)	0/23 (0%)		0/41 (0%)			0/23 (0%)
*** ***	***Total locality IF***	***0/6 (0%)***	***1/43 (2*.*3%)***	***0/23 (0%)***	***-***	***0/6 (0%)***	***19/101 (18*.*8%)***	**20/179 (11.2%)**	***0/6 (0%)***	***0/43 (0%)***	***0/23 (0%)***	***-***	***0/6 (0%)***	***25/101 (24*.*8%)***		**25/179 (14.0%)**			**3/101 (3.0%)**
	Bransan	-	0/3 (0%)	0/23 (0%)	-	0/1 (0%)	-	0/27 (0%)	-	0/3 (0%)	0/23 (0%)	-	0/1 (0%)	-		0/27 (0%)			-
***NI ***	Mako	-	-	0/26 (0%)	-	-	-	0/26 (0%)	-	-	0/26 (0%)	-	-	-		0/26 (0%)			-
	Segou	-	-	0/24 (0%)	-	0/3 (0%)	-	0/27 (0%)	-	-	0/24 (0%)	-	0/3 (0%)	-		0/27 (0%)			-
*** ***	***Total locality NI***	***-***	***0/3 (0%)***	***0/73 (0%)***	***-***	***0/4 (0%)***	***-***	**0/80 (0%)**	***-***	***0/3 (0%)***	***0/73(0%)***	***-***	***0/4 (0%)***	***-***		**0/80 (0%)**			**-**
	Mereto	1/6 (16.7%)	0/4 (0%)	-	1/29 (3.4%)	-	3/13 (23.1%)	**5/52 (9.6%)**	1/6 (16.7%)	1/4 (25%)	-	6/29 (20.7%)	-	0/13 (0%)			**8/52 (15.4%)**		**0/13 (0%)**
** **	**Total**	**1/12 (8.3%)**	**1/67 (1.5%)**	**0/96 (0%)**	**1/29 (3.4%)**	**0/15 (0%)**	**37/212 (17.5%)**	**40/431 (9.3%)**	**1/12 (8.3%)**	**1/67 (1.5%)**	**0/96 (0%)**	**6/29 (20.7%)**	**0/15 (0%)**		**59/212 (27.8%)**			**67/431 (15.5%)**	**12/212 (5.7%)**

**Fig 2:**
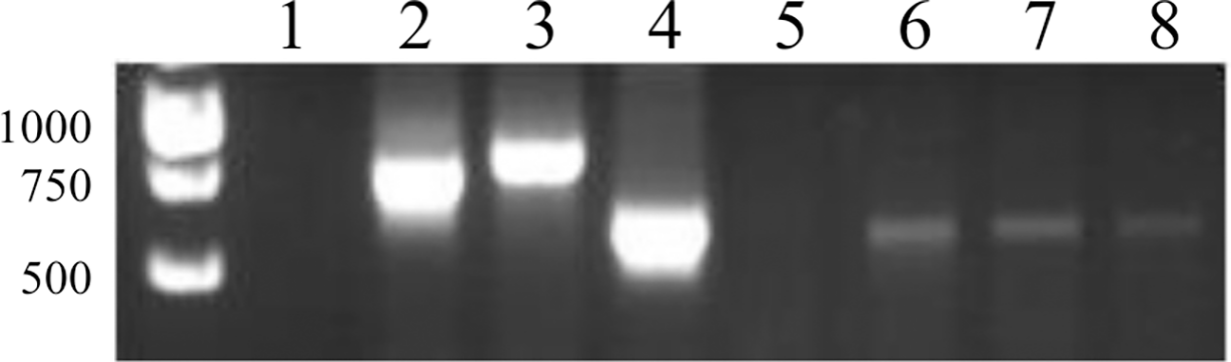
Results of the nested PCR assay used for the identification of *Leishmania* parasites in rodent samples and reference strains. Lane 1, negative control; lane 2, *L*. *infantum* (MHOM/BL/67/ITMAP263); lane 3, *L*. *tropica* (MHOM/SU/74/K27); lane 4, *L*. *major* (MHOM/SU/1973/5ASKH); lane 5, sample 3119 (negative); lanes 6 to 8, samples 3394, 3441 and 3493 (positive).

Comparison of the amplicon sizes obtained for the *R*. *rattus* samples and the *Leishmania* reference strains of the Old World led to the identification of *L*. *major* in all positive *R*. *rattus* samples (**Fig 2**). However, the diagnosis could not be confirmed by direct sequencing of such amplicons. This can be explained by the high heterogeneity of the minicircle kDNA sequences. Each band on agarose gel could include different amplicons of equal size for a single *Leishmania* species. Diagnosis with more specific, but less sensitive molecular methods based on detection of *Leishmania* ITS1, HSP70 or mini-exon, as well as other methods based on kDNA amplification, also failed (**S2 Fig**). Therefore, the amplicons of all the positive nested PCR samples were cloned. The cloning was successful for seven samples (43 clones were successfully analysed), as well as for two *L*. *major* reference strains (Friedlin and 5ASKH) and one *L*. *major* clinical isolate (LC-DKR) (26 clones analysed for these three *L*. *major* strains) (**S1 Table**).

Twenty of 26 sequences from reference strains displayed homologies with sequences in databases. Interestingly, six sequences of excellent quality of the reference strain clones (for instance, the Friedlin-x and LC-DKR-p clones) could not be extensively matched with database sequences (<80 nucleotides), implying that kDNA sequences of *L*. *major* are not yet exhaustively present in databases. In addition, six sequences from the reference strains displayed homology with kDNA sequences from other strains of the same species (for instance, the Friedlin-v clone with the LC-DKR-v clone, with 84% similarity on 572 bp) or even of other species but with low similarity (the Friedlin-d clone with *Leishmania braziliensis*, with 91% similarity on 53 bp, see S1 Table), in accordance with the idea that each *Leishmania* strain has different minicircle kDNA classes that could be either species-specific or shared with other species [54,55].

In the same way, 20 of 43 field sample clones displayed no similarity with the database sequences nor with the reference strain sequences. Nevertheless, two field sample clones (clones 3394–11 and 3394-11c) showed sequence similarity (more than 70% identity with high probability and high score) with *L*. *major* in databases (e.g. LMJLV39_SCAF000371, 73% identity in TriTrypDB on 630 bp) and 21 field sample clones, obtained from five different samples (3441, 3851, 3167, 3192, 3767) showed strong sequence similarity to the sequence of LC-DKR-u (Senegalese *L*. *major* control strain) (**S1 Table**). Interestingly, no clone from field sample showed similarity with sequences of clones of the other two reference *L*. *major* strains used in this study, 5ASKH (isolated in Russia) and Friedlin (isolated in Israel). In conclusion, five of the seven positive field samples tested by cloning could be identified as similar to the Senegalese *L*. *major* strain. Therefore, based on the amplicon size identity and sequence comparison, it was assumed that all the other positive samples belonged to the *L*. *major* species. Altogether, these results designated *R*. *rattus* as carrier of *L*. *major* parasites.

*Leishmania* diagnosis was also performed using DNA isolated from the ear of 64 rodents trapped in the south, chosen among nested PCR positive and negative samples using spleen DNA. Overall, specimens of different species, native or invasive, and the three locality categories (not invaded, recently invaded, and of long-established invasion) were represented. All tested ears were negative.

It is worth mentioning that for some samples (about 7%, generally not *R*. *rattus* samples), PCR products that were clearly smaller than the expected amplicon size for any *Leishmania* species (about 200-300bp, **S3 Fig**) were also observed. Sequence analysis of these amplicons showed similarities with GenBank rodent sequences, suggesting the unspecific amplification of the rodent host genome (**S3 Fig**).

### *Trypanosoma lewisi* diagnosis

All rodents trapped in the north of Senegal (*Ma*. *erythroleucus* and *M*. *m*. *domesticus*) were negative for *T*. *lewisi* (FRET-based real time PCR method).

In the south including Mereto, 59 *R*. *rattus*, six *M*. *m*. *domesticus*, one *A*. *niloticus*, and one *Ma*. *erythroleucus* (spleen samples were positive for *T*. *lewisi* (mini-exon real time PCR method) (**Table 2**). Except R. rattus, all the rodents were from Mereto. No infection was detected in NI localities. In *R*. *rattus* samples (the main host), *T*. *lewisi* prevalence was similar in LI and IF localities (*F*_1.199_ = 1.11, *p =* 0.292).

None of the 13 *R*. *rattus* spleen samples from Mereto was positive for *T*. *lewisi*.

Some *R*. *rattus* samples were positive for both *L*. *major* and *T*. *lewisi*: 9/98 (9.2%) in LI localities, and 3/101 (3%) in IF localities (12/199, 6.0%, for both locality categories) (**Table 2**). In LI localities, *L*. *major* prevalence was higher among *R*. *rattus* individuals that were also infected by *T*. *lewisi* than among those that were not (26.5% vs 9.4%, GLMM *F*_1.199_ = 4.74, *p =* 0.029).

## Discussion

### Leishmania major

The nested PCR allowed detecting 40 positive spleen samples with amplicons of molecular weight similar to *L*. *major* species. All tested ears were negative, suggesting that ear specimens from rats with or without skin lesions should not be used for *L*. *major* detection. Direct sequencing of the positive nested PCR to confirm diagnosis was previously used by other authors [56] or by our team [18]. However, the high heterogeneity and number of minicircle kDNA sequences existing at strain level can prevent the direct sequencing. Indeed, the polymerase may amplify only one minicircle target or several at the same time, depending on the *Leishmania* strain and the experimental conditions. When this happens, as in our study, cloning is necessary prior to sequencing and species identification. Sequencing of field sample clones allowed finding homologies with cloned sequences of a *L*. *major* strain isolated from a Senegalese patient (LC-DKR). The high-throughput sequencing of minicircle PCR products developed on vectors and *Leishmania* strains from the New World could overcome these issues [55].

Finally, 17.5% of all trapped *R*. *rattus* individuals and only two native rodents were positive for *L*. *major* in the south of Senegal (including Mereto), whereas all rodents trapped in the north were negative.

Gerbils are the main *L*. *major* reservoir: *Rhombomys opimus* (great gerbil) in Central Asia, *Psammomys obesus* (fat sand rat) in Middle East, and *P*. *obesus* and *Meriones* sp. in North Africa [57,58]. *L*. *major p*revalence in these species varies from 0 up to 70%, according to the diagnostic method and the season [59]. More recently, *M*. *m*. *domesticus* (42.9% in [60] and 7.1% in [56]) and *R*. *rattus* (12.5% [56]) also have been identified as carriers in the Middle East. So far, in West Africa, *L*. *major* was detected in native *Mastomys* spp., *G*. *gambianus* and *A*. *niloticus* [21,26,27,61]. In Senegal, the prevalence determined by tissue culture was about 7.3% in *Ma*. *erythroleucus*, 5.8% in *G*. *gambianus* [26], and 2.7% in *A*. *niloticus* [62]. However, no parasite by microscopic observation nor typical cutaneous lesion was identified in any rodent species in this country [63]. Among the previous studies conducted in Senegal in the 1980s, very few *R*. *rattus* individuals were found to be carriers, because this rodent species was not sampled at that time in the studied regions (villages near Dakar and Thiès, in the centre of Senegal) [21,22,27,64,65,66,67,68]. Thus, rats were not suspected to play a role in *L*. *major* transmission in Senegal.

*R*. *rattus* has been probably introduced in Senegal from Europe, where *L*. *major* has never been detected [11]. CL is known to be endemic in Senegal since 1933 [64]. Our data showing *R*. *rattus* individuals positive for *L*. *major* in Senegal suggest that invading black rats have acquired the pathogen after their introduction. The finding that native *Mastomys* spp. and *A*. *niloticus* individuals, which were previously described as *L*. *major* reservoirs in Senegal, were not infected in our study could be explained by many factors, such as the ecology of native rodents compared with that of *R*. *rattus*, a different susceptibility between native and invasive rodents, *Leishmania* ecology (transmission generally occurs within a very limited area, micro-foci [71]), *L*. *major* life cycle characteristics (wild zoonotic cycle) and/or the ecology of *P*. *duboscqi* (its main vector) that is more abundant in fields and farming areas than inside villages and towns [69]. A sampling in the surrounding of the villages invaded by *R*. *rattus* is needed to confirm the presence of a native wild rodent reservoir. Particularly, the screening of *L*. *major* in native gerbils, which are common in non-commensal habitats of southern Senegal (e.g., *G*. *gambianus*, *G*. *guineae* and *Taterillus gracilis* [46]), could bring information.

On the other hand, no rodent was positive for *L*. *major* in the northern region invaded only by *M*. *m*. *domesticus*. This could be explained by the absence of *L*. *major* in this region, or by a difference in susceptibility between mice and rats. No comparative experimental study was available, but several epidemiological works showed more cases of *L*. *major* infection in *R*. *rattus* than in *M*. *m*. *domesticus* [56,70]. In Mereto, the village invaded by both *R*. *rattus* and *M*. *m*. *domesticus*, 16.7% (n = 1/6) of *A*. *niloticus*, 3.4% (n = 1/29) of *M*. *m*. *domesticus* and 23.1% (n = 3/13) of *R*. *rattus* individuals were positive for *L*. *major* (**Table 2**), supporting again the hypothesis of a higher susceptibility of *R*. *rattus* to *L*. *major*, compared with *M*. *m*. *domesticus*.

### Trypanosoma lewisi

We found the highest prevalence of *T*. *lewisi* among *R*. *rattus* in the south of Senegal including Mereto (29.6%). Two native rodents (n = 2) and six mice were also positive, but only in Mereto where rats and mice coexist. No infection was found in the north.

*T*. *lewisi* prevalence in *R*. *rattus* is consistent with several previous works often based on microscopic observation rather than on molecular diagnostic tools. *T*. *lewisi* has been identified in the Rattini group of rodents on all continents: Europe [32,46,65], Africa [33,34,35,36,72,73], Middle East [74], Asia [75,76,77,78,79,80,81], North [82], Central [83] and South [84,85,86,87,88] America, Hawaii [89], New Zealand [90] and Australia [91], where it caused the extinction of two native host rodent species in Christmas Island [6]. In Niger, *T*. *lewisi* prevalence is higher in *R*. *rattus* (71%) than in native rodents (6%) [33]. In the other cited studies, the reported prevalence ranges from 4.6 to 82.3%, and varies according to the season, sex and age of rodents [86,90]. Prevalence may also vary according to the tissue chosen for diagnostic. Indeed, spleen have been successfully used in previous studies because it is highly blood-supplied tissue [33,35]. Nevertheless, it may lead to underestimate prevalence levels of the parasite that poorly enters organs, compared to blood samples [37]. *T*. *lewisi* distribution and lack of positive samples among native rodents in our study strongly suggest that Senegal was *T*. *lewisi*-free before the arrival of *R*. *rattus* from Europe. The GLMM analysis did not find any significant difference in *R*. *rattus* prevalence between LI and IF localities, possibly due to the large variability at IF localities (**Table 2**). Indeed, *T*. *lewisi* was not detected in two IF localities (SOU, BOU: **Fig 1**), whereas its prevalence in the other two (KED, BAN, **Fig 1**, **Table 2**) was comparable to that in LI localities. These differences may be related to the variable *R*. *rattus* introduction times in IF areas. Indeed, Soutouta (SOU) and Boutougoufara (BOU) are marginal rural localities that have undoubtedly been colonized very late by *R*. *rattus*, because their connection to commercial networks dates from 2007 [92,93]. Conversely, Kedougou (KED) and Badi Nieriko (BAN) were colonized by *R*. *rattus* in the 1990s [41]. The prevalence heterogeneity at the IF is consistent with the hypothesis of *T*. *lewisi* introduction by *R*. *rattus*, and reflects classical enemy loss [49] in localities that have been very recently invaded. *T*. *lewisi* infection has been associated with a large mortality rate in non-Rattini species [35], which could lead to under-estimation of parasite transmission in these hosts. If this parasite affects severely native rodents, its “spill-over” [49] from the host that introduced it would confer to rats a strong advantage at the IF.

Like for *L*. *major*, the absence of *T*. *lewisi* infection among rodents in the north may suggest a lower sensitivity of mice. Mice and rats are both used as laboratory models for *T*. *lewisi* infection, but mouse experimental infection sometimes fails, while rat infection is more reproducible [76,94]. At first glance, the data obtained in Mereto challenge this idea: in Mereto 21% of *M*. *m*. *domesticus* and two native rodents (*A*. *niloticus* and *Ma*. *erythroleucus*) were infected, while no *R*. *rattus* (0/13) was positive for *T*. *lewisi*. *Trypanosoma musculi*, another trypanosomatid species very close to *T*. *lewisi*, could be responsible for the infection [95]. Indeed, *T*. *lewisi* and *T*. *musculi*, morphologically indistinguishable, were characterized on the basis of host specificity, rats being preferential hosts for *T*. *lewisi* and mice for *T*. *musculi* [95]. Moreover, these two species appear also difficult to distinguish on a molecular point of view [95,96]. We first tested the molecular methods used in our study for *T*. *lewisi* diagnosis on the reference strains of *T*. *lewisi* and *T*. *musculi* (Partinico II strain kindly provided by Pr Philippe Vincendeau and Mrs Pierrette Courtois). The gel electrophoresis banding patterns and the sequencing did not allow distinguishing these two parasites (**S4A and S4B Fig**). Secondly, the only published PCR protocol able to differentiate *T*. *lewisi* from *T*. *musculi* [96] was also tested. The results obtained in our assays were non reproducible, non-specific and not sensitive enough (**S4C and S4D Fig**). Furthermore, the only two sequences available for *T*. *musculi* in Genbank database make difficult the design of a new specific and more sensitive molecular diagnostic method. Therefore, at this stage, we cannot deduce which of *T*. *musculi* or *T*. *lewisi* is the parasite responsible for infection in Mereto positive mice.

The prevalent pattern of *T*. *lewisi-*positive *R*. *rattus* co-infected with *L*. *major* in LI suggests positive interactions between the two parasites. Previous studies showed that *T*. *lewisi* could weaken the immune system of its host and thus favour the acquisition of other infections, such as *Toxoplasma gondii* [97,98] or *Cryptococcus neoformans* [99].

### Putative consequences on human health

Data about *L*. *major* distribution in Senegal are still limited, with the exception of the Dakar region [23] and nearby localities [21,22], where it is endemic. To date, no data is available about human cases caused by *L*. *major* in the north nor in the south of this country. Classically, the transmission of *L*. *major* takes place essentially in rural environments, peridomestic and farming areas, where the burrows are, with an epidemiological cycle involving native rodents as main reservoirs [18,21,100]. We can imagine that a second transmission cycle involving *R*. *rattus* might occur predominantly inside villages and indoors. The increasing presence of *R*. *rattus*, which has a behaviour strongly linked to human activities and can move and settle in new territories, engenders a risk of emergence of new spots of human CL. It would be interesting to determine the number of cases and spatial distribution of human CL in Senegal, and to assess the available means and knowledge for its diagnosis.

Only nine cases of *T*. *lewisi* infection in humans have been reported in the world, in Malaysia [101], India [76,102,103,104,105], Gambia [36] and Thailand [106]. Patients were often immunologically weak infants, living in poor hygiene conditions, and in close contact with contaminated rats in and around houses [36,76,101]. Symptoms were generally mild, except in one child [105]. The diagnosis was mostly based on the morphological identification of *T*. *lewisi* by microscopic examination of blood drops [76,101,102,103,104], and was rarely confirmed with molecular tools [36,105,106]. However, human infections could be underestimated, because the identified patients lived far from health centres and *T*. *lewisi* infection is usually associated with non-specific and transient symptoms (fever, lethargy, anorexia) [30]. Very few epidemiological data on humans have been published and no routine and specific serological tests are available yet [107]. Nevertheless, 12 of the 187 farmers tested were serologically positive in China, without any apparent symptom [108], reflecting the existence of asymptomatic carriers. The risk for immunologically weak people, such as patients with AIDS, remains to be evaluated. Given the high prevalence of *T*. *lewisi* in rodents in the south of Senegal, it could be interesting to assess the seroprevalence in humans in this region. This could be performed by the international network for atypical human trypanosomiases that has been set up in Africa [107].

In summary, we identified the black rat *R*. *rattus* as a potential reservoir for *L*. *major* and *T*. *lewisi* in the southern part of Senegal. These two infections appeared to obey to two different models. The invader *R*. *rattus* could have been be locally infected by the endemic parasite *L*. *major*, and is potentially more susceptible than native commensal rodents. Conversely, *T*. *lewisi* infection could have been introduced in Senegal by *R*. *rattus*, but seems to be poorly transmitted to native rodents by *R*. *rattus*, although this point remains to be investigated. The high prevalence of both parasites in *R*. *rattus*, which is anthropogenic and relentlessly gaining new territories, could increase the risk of transmission/emergence of new foci of human CL in urban areas and of sporadic cases of human trypanosomiasis.

## Supporting information

S1 Table BLAST analysis of the sequences obtained by PCR amplification for *Leishmania* detection. For *Leishmania* diagnosis, the nested PCR method to amplify the minicircle kinetoplastic DNA (kDNA) variable region described by Noyes et al. [51] was used, and then amplicons were separated by electrophoresis before cloning. * BLAST results were retained only if they corresponded to the sequences of reference strains or to sequences of isolates described in peer-reviewed articles and well characterized in terms of species, origin and hosts, in order to avoid misidentifications. ** When the results obtained with one database were highly significant and with high score whereas low similarity was found with the second one, only the highly significant results are provided.
(DOCX)

S1 Text Sequences obtained by PCR amplification for *Leishmania* detection. (DOCX)

S2 Text Sequences obtained by the mini-exon PCR amplification for *Trypanosoma lewisi* detection. All the sequences presented were obtained from *R*. *rattus* samples.
(DOCX)

S1 Fig Study of the FRET-based real time PCR on 18S rRNA used for *T*. *lewisi* detection. (A) Cp values of amplification of a *T*. *lewisi* DNA scale and of other trypanosomatids. (B) Efficiency curve. This method was chosen for its robustness and sensitivity (<9.5fg/μl). However, this PCR was not specific for *T*. *lewisi*, but detected also other trypanosomes as well as *Leishmania*.
(TIF)

S2 Fig Negative results obtained with other molecular methods tested to confirm *Leishmania* diagnosis. Field samples were chosen among samples that were positive with the nested PCR used for the *Leishmania* diagnosis and tested with other molecular methods. The sensitivity of these protocols was not sufficient to confirm the diagnosis made with the nested PCR on kDNA minicircles.W stands for water (negative control). Primers were the following: ITS1: LITSR, ITS1R [109]; nested ITS1 step 1: LITSR, LITSV, step 2: LITSR, L5.8S [110]; HSP70: forward [111], HSP70ant [112]; mini-exon: Fme, Rme [113]; ITS2: LGITSF2, LGITSR2 [114]; rDNA: rDNA-10F, rDNA-14R [115]; kDNA: L.MC-1S, L.MC-1R [116]; kDNA (real-time PCR HRM): MLF, MLR [117].
(TIF)

S3 Fig Unspecific amplification of rodent DNA with the nested PCR on kDNA minicircles. (A) Exampe of electrophoresis gel showing unspecific amplification obtained from a few field samples (7%) with the nested PCR on kDNA minicircles (samples marked with *, lower bands). (B) Example of sequence obtained from the sample CB3834 and presenting 85% homology on 406b with mouse DNA sequences found in public databases (for example GenBank AL772311.19). Overall, seven amplification products from seven field samples were directly sequenced and showed homology with rodent DNA.
(TIF)

S4 Fig Tests for *T*. *musculi* as another possible interpretation for the *T*. *lewisi* positive results in *Mus musculus domesticus* samples from Mereto. (A) Electrophoresis gel of the *T*. *lewisi* mini-exon PCR performed on *T*. *musculi* and other reference strains. Our *T*. *lewisi* diagnosis was not strictly specific and could amplify *T*. *musculi*. (B) Sequencing of the mini-exon PCR product from *T*. *musculi*. There were too many variability and too few data in databases to differentiate *T*. *lewisi* and *T*. *musculi* on the basis of mini-exon sequencing. (C) Electrophoresis gel of amplification of different reference strains with *T*. *musculi* kDNA maxi-circles PCR (upper part). The four *T*. *musculi* samples were extracted independently from the same blood sample of an infected mouse. Primers used were TM1F, TM1R [96]. In the lower part, amplification of the same samples with the 18S rRNA PCR showed that the *T*. *musculi* kDNA maxi-circles PCR seemed to be not very sensitive. (D) Electrophoresis gel of *T*. *musculi* kDNA maxi-circles performed on field samples (upper part: rat samples; lower part: mouse samples). This PCR gave frequent (33/66, 50%) non specific amplification, probably from rodents DNA. We obtained no band of size corresponding to specific amplification.
(TIF)
