# Supplementary material for: Leishmania major and Trypanosoma lewisi infection in invasive and native rodents in Senegal
Source: PLoS Negl Trop Dis. 2018 Jun 29;12(6):e0006615. doi: 10.1371/journal.pntd.0006615 (PMC6042788; doi:10.1371/journal.pntd.0006615)
Supplement: S1 Text — (DOCX) [file pntd.0006615.s002.docx]

**Text S2. Sequences obtained by PCR amplification for *Leishmania* detection.**

**Sequences submitted to GenBank**

**>5ASKH-1b.scf / GenBank MG209676**

TAAAATAGGGCCGGGTGGGTCGGGGGATTGAGCTGGTCGGCCTTTTTATACGGGCTAGTGGCTTGGATTTACACGTCCTAACTTTTGGCTTTGATTTTTGATATTTAATTCTGGGTTTGAGGTGGTAGTGGTTGTGTGGGTTGTGTGGGTTGGATTGGATGCTTTGTTTTGTGGGTATAGGTTGTAGTCTTTGTAGGTGGGTCGTGGGGCGCCTTGGGGGTATCTGTTGATGCCTTAGGGGGTGGCCCGGGTGCCTTGGTAGGGATGCCTTTAATATTGATATTAAAGGACTTAGAACCTTTATTAGGTAACTTGCTGGTTTGACAGTGATATTAATGGTAGTTAATATTAGTGTCTTATAAGTTGTTATAGATTATTGTATATATTACGTGTATTCATGTTATCTTTATCGGTGTGAGTGCTAATATTTCGTAAATTTGGCTTATATAAAATGCTAGAGTGTAAGACTACATAATAGCCTTATAAATATTGTTGTTGGATTTCATTGGAGGTGTGAATTTGTGTTGAAAAAGCTAAAAATGGGTCCAAAAT

**>5ASKH-1c.scf / GenBank MG209677**

TGTAAAATAGGGCCGGGTGGGTCGGGGAATTGTGCTGGCCGGCCTGGACATACGGGATTGGCGCGATCTTTTTGGTGTTTTGAGGTTAAGTCTTTTTTATTCGGGGTTTAGGTTTAGATTTGTAGTTTGAGTTGTGTTGTTGGTTGTAGGAGTTTGGTTTCTTGGGTTGAATTGGTTTTGATTTTGATTGGGTTGGAGGGGTGTAGGTATTGGTGCAGTGCTAGATATCGATGGATTAGAGTGTTAGTGGGGTGGGAATTGCTTTACTTTAATATTTATATTAAGGGAGGGGTTATGTAGCCTTAGTGTGTATGGTAAGGCGTCTTACTAGTGGGTAATATAAGGGTTTGTATAACTAGTTTATTTCGATATATTACAATTCTATAGTTTAATCTTCAATAGTTGCTAACTTAGATACTAAACGTTTTACCTGGTTAGATTACTAAAGTATCATGATATAGTTATCTGTTGTTTGGTGTTGTTAGGGTTCTCATTTATTTGGTTC

**>5ASKH-2.scf / GenBank MG209678**

GGGGGTTGGTGTAAAATAGGGTCGGGTGGGAGGGGAATTTGCTGTACCTGCTAGATTATACGTGTGGAGTGGCTTGTTTTCGGCCGGTTGAGACTTTGATCTTGATTTACAATGGGGGTGGGCGTTGTTGTGGGGGTTATATGAGTTCGTGTTTGGATTGGGTAGGTGTTAGCTGATCGAGGCTACTCTGCGTGGGATATGAGGGTGGTGTGGGAGTTGGTCTTTGGGGGTATACGTGACCATGTGGGTCTTAGACGTGGGGTGAGGTTTGATTATTAGCTTTATTCTATATATTGATATGTAACTGGATACTACATAGAGTTTGGGTAATAACATTGTAATTGATAGTATTAAGATTATATTGGTATAGTTACTAGTATTCTTTTACTCATTCTATTCTATTTTTATGTTAAGCTCTGTTAACTGATACGTACTGATACTAAAATATGCGTAGTACTATAATGCTTACAGCTGAGTTATTAGTGGCTATATTGTAAGTGCGGTTTGGATTTTGAGTATTGTCTTGAAAATGGTCAGAAAATGCCTAAATTCCCAAACTTTTCTGGTCCCGCGGGTAGGGGCGTT

**>5ASKH-2a.scf / GenBank MG209679**

CCGGGCGTTTGGGCTGTATGGGGCTGGCGGCGGGTTTTCGACTTTGTTGGGATTTGTGACTGATATTTGAGCTTTAAACGACGACTTTGTGGTTTTGAGGATTGGTTTATTGTAGCGGGCTTTGTATGATGAGATTGGTATTGGAGTGATAGAGGGCGTGGGTTATAATTATGGATAATTGGTATAAAGTTGGTGTGGTGGTGTTACATGTGGGTTCTCACACTTAATATATAGAATATTCGTAATATTTAAATAATGGGGTTGTAAAGTTATTGTTACGCTAGATTTAGTTAAATAGATTAGTTAGTATAATTACGTGAAGATAACTGATATTACTTATATGCTATAAATACTATGTTTAATATTATATAGAACTTAGATGTGAACGATTGTTAGTATGTAATGCCTTACCTCTTACAAGATATGAGATATGTTTTACGTAGCTATGGGTGTTTGGATATGGTGACACGGGGTCAAAAATCCTAAAAAATGGGTCCAAAATCGAAACTTTTCTGGTCCCACGGGTAGGGGGGTTCTGCGA

**>5ASKH-2f.scf / GenBank MG209680**

TAAAATAGGGCCGGGTGGGGATGGGAATTCTCTGGAGTGGGCTGGGTATACGGGTGGAGAGGCCTACTTTGGAGAGTTTTGACTTTTATGGGTTTCGATGGCTGCTTTGTGTTTGATTTTAGTGTTGGGGTGACGGTTCTGTAGTTTTGTTATGAGTGCTGGTTCAGGTTGCTAAGAGGGGTTGTGTTTATTGGTGCTACGCCTTGGGGGTGGGTGTTTGGCACCTTGTTGTATGTGTGGGAGATAGCCTGGGAACTTAATATACAGAATATATTATATATTAGTGGGTAAGCGTTTAGGGTGTAGGTTAGTAGAGTTATAATTAGTGATTATAATGGTTATTGATAAGGTAATATTACGTTAATTTATACTGTAATCTTTGTTCTAATTGTGTTATTGCTCAGTTAGATGTTATTTATAATTAATGATACTGGAGGCATACAACAATCTCTAGTATTATAATAGTCTTTGCAGGTGTTGGATTTGTGTGGGGCGTTTGGGGGAATTCGTGAAACATGAGCGAAAAATGGCCGAAAA

**>5ASKH-3.scf / GenBank MG209681**

GCGGGATTACTGGGGGTTGGTGTAAAATAGGGCCGGGTGGGTCGGGGGATTGAGCTGGTCGGCCTTTTTATACGGGCTAGTGGCTTGGATTTACACGTCCTAACTTTTGGCTTTGATTTTTGATATTTAATTCTGGGTTTGAGGTGGTAGTGGTTGTGTGGGTTGTGTGGGTTGGATTGGATGCTTTGTTTTGTGGGTATAGGTTGTAGTCTTTGTAGGTGGGTCGTGGGGCGCCTTGGGGGTATCTGTTGATGCCTTAGGGGGTGGCCCGGGTGCCTTGGTAGGGATGCCTTTAATATTGATATTAAAGGACTTAGAACCTTTATTAGGTAACTTGCTGGTTTGACAGTGATATTAATGGTAGTTAATATTAGTGTCTTATAAGTTGTTATAGATTATTGTATATATTACGTGTATTCATGTTATCTTTATCGGTGTGAGTGCTAATATTTCGTAAATTTGGCTTATATAAAATGCTAGAGTATAAGACTACATAATAGCCTTATAAATATTGTTGTTGGATTTCATTGGAGGTGTGAATTTGTGTTGAAAAAGCTAAAAATGGGTCCAAAATCCAAACTTTTCTGGTCCCGCGGGTAGGGGCGTTCTGCGAAA

**>Friedlin-b.scf / Genbank MG209686**

TACTGGGGGTTGGTGTAAAATAGGCCGGGTGGGTCGGGGAAATTCCTAGCGGGGTGGATTTATATGGGTTCTGAGGCCTTGTTTGTAATGTGTTGGAAATTCTCTTTAAATTTTGTCTTTAAAAGTTTGATTTTGTGGGGTTGTGAGTTTTGTTGGTGTCGAGTTGGTGGTGATAGCGCTTTGGCGGGGTGTGGGGTTGGTGATGGTGGTTGTGGGGTTGTGATGAGCTTTGGAGTCAGGGCGAACTTGTGGGGTGGGGTATATCGTGGGACACAAACTTCCTTTACTTTAATATTGATATTAGTTGGTACCCTGGCATAGGGAGGTTAAGAGCACATAGACGCATGGTAATATAGTGGTAAAGCATCTGTATATTCACGTATTGTATTATTTAAACTTACTTTACATAGATCTTTGCGGTCTTTGCGTTACAACTTAGTGTTACGGATATGTTCGTGGTGTACTATGTATACTTACCGTATACTAAACTGTTTATTGGTGATACAAGGTTGTATGCGTGGATTTGGTGATTTTTGTTAAAAAGTCAAAAAATAGGGCA

**>Friedlin-d.scf / GenBank MG209685**

TACTGGGGGTTGGTGTAAAATAGGCCGGGTGGGTCGGGGAATTCTTGATTTTTTGGGTGGGATCCGCGGGAACGCCGGGCTTGGAGGGCGCATTTTGGGATACTGGGATTTTGATGGTTGGGGCTAGTGGGGTTTGGCGCATTTCTGGGCTTGTGTTGGGTCGACTGTGTGGAGGCTGAGGCGGTGTGAGTGGTGTGGTTCCTGGTGGGTATGTGGGTGTGGCCAGCCTTGGAGGTGGGGGTTGGGTTATGGTGGGTGGTAAGTGTTATATGGGCTCTTTGGGGTTTGATATATGCTTTATTCGGTATATTAAGGCTTTGGGGACGGGTTTGGAGCCTGGTCCTAAATTGAATGTCTTTGCACTGGATATAGTAGCTATATAAATAGGTAAGCGTATTTATAGTTTGTATATATTTGGTAATCTGTAATTTAAAGTTTACTCTATCTGTATTGCATTGTTTGTATCTGTAACGTTATAATTTAGTGCTAATAGATATAGTATTTAACTACTCTAAACTCATACTAGATTTGCAGAACGTTCTAATTTTCTTGGAATTTAGCTAAAAATAGGGCAAT

**>Friedlin-v.scf / GenBank MG209687**

TACTGGGGGGTGGTGTAAAATAGGTCGGGTGGGTCGGGGAATTGCGCTGGCCGCCCTGGATCCGCGGGGTTGGAGGCCTGTTCGAGGCTGGGCTGGATGGTACGGAGGTTTGTGACTTGTTTTTGGACTTTAACTTGATGTTTGGTGTGGGGTACGACGTTGTGGGGGTTTGAGGTTGGAGATTCTCGCTGATACTTGTGGGGCTTCAGGGTGTTAGTGGTTCGGGCTTGGATTGACTAGGGTGTAGTGGGGAATGGGTCTGGTGGTTAACCAGAGGGTGTGGGGTTATATACAGAATAAGTTATATATTAACTTATGATGATAATATGCCGGTTAGGCGTAATGGCTGAGGGAGGATAGAATAACGACCGATGCATATTAGGTGTATAGACTGTGAACCGGATGAACAGTTTATACATATTACTCTATAGTTATATGTAGTTTTTCTATACTTTGCGCTATTTACTGGGGGTTGGTGTAAAATAGGGGCGGGTGGTCCAGGTCCGGCTTGAACTGGCCTCCTGGTGGCTTGAATTCGCGTGGCTGGTCTTTTCTGGTGGGG

**>Friedlin-x.scf / Genbank MG209688**

CTGGGGGTTGGTGTAAAATAGGGGCGGGTGGTTCAGGTCCGGCTTGaACTGGCCTCCTGGTGGCTTGAATTCGCGTGGCTGGTCTTTTCTGGTGGGGTTGGGTTTTATACTTGGAATTTGGGCTTTAACGCAGCTTGTTTAGGGGCGTTCTGCGAGACTGGCCTCCTGGTGGCTTGAATTCGCGTGGCTGGTCTTTTCTGGTGGGGTTGGGTTTTATACTTGGAATTTGGGCTTTAACGCAGCTTGTTTAGGGGCGTTCTGCGA

**>LC-DKR-ad.scf / GenBank MG209689**

TACTGGGGGTTGGTGTAAAATAGGTCGGGTGGGTCGGGGAATTGCGCTGGCCGCCCTGGATCCGCGGGGTTGGAGGCCTGTTCGAGGCTGGGCTGGATGGTACGGAGGTTTGTGACTTGTTTTTGGACTTTAACTTGATGTTTGGTGTGGGGTACGACGTTGTGGGGGTTTGAGGTTGGAGATTCTCGCTGATACTTGTGGGGCTTCAGGGTGTTAGTGGTTCGGGCTTGGATTGACTAGGGTGTAGTGGGGAATGGGTCTGGTGGTTAACCAGAGGGTGTGGGGTTATATACAGAATAGTTATATATTAACTTATGATGATAATATGCCGGTTAGGCGTAATGGCTGAGGGAGGATAGAATAACGACCGATGCATATTAGGTGTATAGACTGTGAACCGGATGAACAGTTTATACATATTACTCTATAGTTATATGTAGTTTTACTATACTTTGCGCTATTTTACTCTAATTTTGCATATGATAACGTAATAAGATAGAGAAACAGATGATGTAACATAACTGTGCGTGTTATATGACTCCTATCGAGCTCAAAATTCAAAAAATACCCCAA

**>LC-DKR-ah.scf / GenBank MG209690**

TACTGGGGGTTGGTGTAAAATAGGGCCGGTGGGGTCGGGAATTCTCCGGAgTGGCCTGGATATACGGGGTTGGAGGCTGGTTTCGATGGGTTCTAATTTTGTGCCTTGATTTTGATCTTTATTTTGGGTGTTTGAGGTCAGATTGCGTGTGAAGATTGAATACTCGGGGGTACGGTGGATGTTAATGGATGTTAATGGTTGTTAATGGCCTCTGTGTTGATAGGTTGAGGTGGGAGTTGTCTTATATGAGTGGCTGGCTCTTTTGATAAGGGTCAGAATTGATATATAGAATAAGTTATATATTAGTTTAGAGCCTTAGAGGGCTTTTGGTACCTGGGCGTAGAGTTAATTAGTAGTGGGTTATTATAAGACGTATTATATCTTTAGTATTACAATAATATTTCTATCTTCTAACTTATAACTTTCTCTATTGTATTGCGCTTATTACCGTGCTGTTACTGTAACGTTGTAATTATAATGTAAATAGTAGATAGAGTTCGAGTGGTTACTGTTGTATTAGAATTTGCGTGGATTTCATAGAATTTGCTCAAAAATGGGGCAAATCCCAAACTTTTCTGGTCCCACGGGTAGGGGCGTTCTGCGA

**>LC-DKR-ai.scf / GenBank MG209691**

ACTGGGGGTTGGTGTAAAATAGGGGCGGGTGGTCCAGGTCCGGCTTGAACTgGCCTCCTGGTGGCTTGAATTCGCGTGGCTGGTCTTTTCTGGTGGGGTTGGGTTTTATACTTGGAATTTGGGCTTTAACGCAGCTTGTTTAGGGGCGTTCTGCG

**>LC-DKR-p.scf / GenBank MG209692**

TACTGGGGGTTGGTGTAAAATAGGGGTGGGTGGTCCAGGTCCGGCTcAAACTGGCCTCCTGGTGGCTTGATTTTGGCTGTGCTGGGTGGGGAACATGTTCTGGCGTTTGTTTATGGAGTTAGCGTTGGGTATTTGGGGTTGGTGATATTGTTGTTTCGTTTTGTGATTGGTGCGAGCTTTGATATTGGGATACATAATTGTTTATGTTCGGGCGTGGGCATGGATACCCGATTGAGCCTGGGGTTTAGGTGTGGGTGGGGTGATGCTGGGGTTAGCTCTTAACATATATTTAATATTAGTATAAAGGGGGATATGTGAGGTTATACCGAAGGTGACGGGTATCACATTAGAATATAGTTATGATATCTGTATAGTTTATGCGTGTTTATTATATTTACAGTTACTATTTATTTGTTTACTCTATGTTTGTTACGTTGCGATATTACTAATAAATTTACTGATATGAAACTTTAATCTAATGTATATAAAATAACTATATAAGACTGACTGTGTTACGTGTGGATTTGAGTTTCGAGACATAAAAAGTCAAAAAATGGGCTAAAAATCCAAACTTTTCTGGTCCCGCGGGTAGGGGCGTTCTGCGA

**>LC-DKR-u.scf / GenBank MG209695**

GCTGGCCGCCCTGGATCCGCGGGGTTGGAGGCCTGTTCGAGGCTGGGCTGGATGGTACGGAGGTTTGTGACTTGTTTTTGGACTTTAACTTGATGTTTGGTGTGGGGTACGACGTTGTGGGGGTTTGAGGTTGGAGATTCTCGCTGATACTTGTGGGGCTTCAGGGTGTTAGTGGTTCGGGCTTGGATTGACTAGGGTGTAGTGGGGAATGGGTCTGGTGGTTAACCAGAGGGTGTGGGGTTATATACAGAATAAGTTATATATTAACTTATGATGATAATATGCCGGTTAGGCGTAATGGCTGAGGGAGGATAGAATAACGACCGATGCATATTAGGTGTATAGACTGTGAACCGGATGAACAGTTTATACATATTACTCTATAGTTATATGTAGTTTTACTATACTTTGCGCTATTTTACTCTAATTTTGCATATGATAACGTAATAAGATAGAGAAACAGATGATGTAACATAACTGTGCGTGTTATATGACTCCTATCGAGCTCAAAATTTCAAAAAATACCCCAAAATTCCAAACTTTTCTGG

**>LC-DKR-v.scf / GenBank MG209693**

TACCTGGGGGGGTTGGTGTAAAAATAGGGTCGGGTGGGTTTCGGGGAAATTTGCGGCTGGGCCGCCCCTGGGATCCCGCGGGGTTGGAAAGGCCTGTTCGAAAGGCTGGGGCCTGGAATGGTACGGAAAGGTTTTTGTGACTTGTTTTTTTGGACTTTAAAACTTGAAATGTTTGGTTGGTGGGGTTACGACCGTTTGTGGGGGTTTTTGAGGTTGGAAGAATTCTTCGCTGAAATACCTTGTGGGGCCTTCAAGGGTGTTTTAGTGGTTTTCGGGCCTTTGGATTTGGACCTAGGGTTGTTAGTGGGGGAAAATGGGTTCTGGGTTGGTTAAACCCAGAAGGGTGTTGGGGGGTTATATTACCAAGAATAAAAGTTAATTATATTAAAACTTAATGGAATGATAAATTATGCCGGGGTTAGGGCGTAAATGGCTGAAAGGGAGGATAGAATAACGGAACCGATGCAATTATTAGGTGTTAATAGACTGTGAACCGGGAATGTAACTAGTTTATACAATTATTACTCATATAGATTATATGTAGCTTTTTATATACTTTGCGCTATTTACTGGGGGTTGGTGTAAAATAGGGGCGGGTGGTTCAGGTCCGGCTTGAACTGGCCTCCTGGTGGCTTGAATTCGCGTGGCTGGTCTTTTCTGGTGGGGTTAGGGA

**>LC-DKR-w.scf / GenBank MG209694**

GTGGCTGGCTTTTCTGGTGATGTTGGGTTTTATACTTGGAATTTGGGCTTTAACGCATCTTGTTTAGGGGCGTTCTGCGAAATTGTGGGGGTTTGAGGTTGGAGATTCTCGCTGATACTTGTGGGGCTTCAGGGTGTTAGTGGTTCGGGCTTGGATTGACTAGGGTGTAGTGGGGAATGGGTCTGGTGGTTAACCAGATGGTGTGGGGTTATATACAGAATAAGTTATATATTAACTTATGATGATTATACGCCGGTTAGGCGTAATGGCTGAGGGATGATAGAATAACGACCGATGCATATTAGGTGTATAGACTGTGAACCGGATGAACAGTTTATACATATTACTCTATAGTTATATGTAGTTTTACTATACTTTGCG

**>3394-11b.scf / GenBank MG209682**

TTGTGGGGGTTTGAGGTTGGAGATTCTCGCTGATACTTGTGGGGCTTCAGGGTGTTAGTGGTTCGGGCTTGGATTGACTAGGGTGTAGTGGGGAATGGGTCTGGTGGTTAACCAGAGGGTGTGGGGTTATATACAGAATAAGTTATATATTAACTTATGATGATAATATGCCGGTTAGGCGTAATGGCTGAGGGAGGATAGAATAACGACCGATGCATATTAGGTGTATAGACTGTGAACCGGATGAACAGTTTATACATATTACTCTATAGTTATATGTAGTTTT

**>3394-11c.scf / GenBank MG209683**

CTGGGGGGTGGTGTAAAATAGGTCGGGTGGGTCGGGGAATTGCGCTGGCCGCCCTGGATCCGCGGGGTTGGAGGCCTGTTCGAGGCTGGGCTGGATGGTACGGAGGTTTGTGACTTGTTTTTGGACTTTAACTTGATGTTTGGTGTGGGGTACGACGTTGTGGGGGTTTGAGGTTGGAGATTCTCGCTGATACTTGTGGGGCTTCAGGGTGTTAGTGGTTCGGGCTTGGATTGACTAGGGTGTAGTGGGGAATGGGTCTGGTGGTTAACCAGAGGGTGTGGGGTTATATACAGAATAAGTTATATATTAACTTATGATGATAATATGCCGGTTAGGCGTAATGGCTGAGGGAGGATAGAATAACGACCGATGCATATTAGGTGTATAGACTGTGAACCGGATGAACAGTTTATACATATTACTCTATAGTTATATGTAGTTTTACTATACTTTGCGCTATTTTACTCTAATTTTGCATATGATAACGTAATAAGATAGAGAAACAGATGATGTAACATAACTGTGCGTGTTATATGACTCCTATCGAGCTCAAAATTTCAAAAAATACCCCAAAATTCCAAACTTTTCTGGTCCCGCGGGTAGGGGCGTTCTGCG

**>3441-2d.scf / GenBank MG209684**

ACTGGGGGGTGGTGTAAAATAGGTCGGGTGGGTCGGGGAATTGCGCTGGCCGCCCTGGATCCGCGGGGTTGGAGGCCTGTTCGAGGCTGGGCTGGATGGTACGGAGGTTTGTGACTTGTTTTTGGACTTTAACTTGATGTTTGGTGTGGGGTACGACGTTGTGGGGGTTTGAGGTTGGAGATTCTCGCTGATACTTGTGGGGCTTCAGGGTGTTAGTGGTTCGGGCTTGGATTGACTAGGGTGTAGTGGGGAATGGGTCTGGTGGTTAACCAGAGGGTGTGGGGTTATATACAGAATAAGTTATATATTAACTTATGATGATAATATGCCGGTTAGGCGTAATGGCTGAGGGAGGATAGAATAACGACCGATGCATATTAGGTGTATAGACTGTGAACCGGATGAACAGTTTATACATATTACTCTATAGTTATATGTAGTTTTACTATACTTTGCGCTATTTTACTCTAATTTTGCATATGATAACGTAATAAGATAGAGAAACAGATGATGTAACATAACTGTGCGTGTTATATGACTCCTATCGAGCTCAAAATTTCAAAAAATACCCCAAAATTCCAAACTTTTCTGGTCCCGCGGGTAGGGGCGTTCTGCGA

**Sequences not submitted to GenBank due to lack of match in databases:**

**>3394-21t.scf**

TACTGGGGGTTGGTGTAAAATAGTCAGGGTGGGATTCCCGAGGATTTTGGGAGATAAAATGATAAAGGTTTGGTGCGGGGTGTTAAGATGGGTGGGTATTTAATTTAAGAATATGATCTAGGTGTGATAGTGTATGAATTTATAAAAATATAATTAACGCAAATGGGGGTGTAAACTGATATAGAAATGTGATAATAAATTTGTGTAAATAATAGTGTGTTAGTGGATGTCATAAAATTAAAATAGTATTTGGGAGGGTAGGAGGGAGGTGTGTGATGGTGGATGATGAAGGGGGTGGAACATAAATATTTGATATATGGTTTAAAACTATAATGGGTAGTGGTGATGTAATGTATATAAAACTAACAACACAAAATACATGGTGAGAGATGACATAGATAGATTATATGAGGTGTATGAATTTATGTTTATAAATATTATAATTAATGATTATTTGGTGGGAGGGGTAGGAGGGAGAGGTGTAGTAGGTGTGAATTACAAAAAAAGTTCGCCGAAAAAA

**>3394-21b.scf**

TACTGGGGGTTGGTGTAAAATAGTCAGGGTGGGATCCCCGGAAAAATTAATGGTGGTGGGTGGTGGTAATATTTACGGTATGGTTATTTTATTTAGTTATGTGAGTTATGTGAGTTCATTATTTTATCTGTTGATTTCGTTTATAGATTTGTTTTGTATATAGATTGTGTGGTGTAGAGAATTAGTGGTTAAATATAATACGAATTAATTATTTTCGTGTGGGAGGGGTGGAGGAGGGTGGTGAGAGATAATTTGTTATTATGGTATGATAGATTAGAGATATAGATGATATAATAATATATAATAATAAACACATAAAATTGGATGAACGGAATAATCAATATGTGATGAGAATAGTGTTATAATTTGTGTTGTAAAATATTATTTAATTAATGTTTGTACGGAGGGGTAGGGATAGGAGGGGTTAGGGTGTAGGGTTAAGAATTATTTTTAATTTTAATTTTACAAATTTGTGGAGGTGGGAGGGGTGAGGGGTGAGGATGTTGAAAATCGGAG

**>3394-2.scf**

TACTGGGGGTTGGTGTAAAATAGTGGATCTTCATAGTGAGCGCACGATAGACCCAGGCACACGAGACAGGAGGGGAGGTTGTGAGAAAGAGACTTGGCAGCGTGTTTAAGGGTGGACAAGGGGCGTTCTGCGACCTAACCCTAACCCTA

**>3394-4.scf**

TCGCAGAACGCCCCTATGCTAAGGGTTTGAACATCATCTTTGAGGAGGATAATAATTATTTTCTCAGTATGACGGACTACTATTTTACACCAACCCCCAGTA

**>3394-21a.scf**

TACTGGGGGTTGGTGTAAAATAGTCAGGGTGATAGAACGAGGGGTAGGAGGTGATATCCTAAATTTTAAAAATGTGGTGGTGTTGATGTTGTTTGTTTGTTTGTTTAGGTGATAGGTGAGGTATGGTGTGGTAGAAGAAGTAAATTTTATTTTAGAATTTGGAAGTGGTGGAGAGGGTAGTAGGTGGGGTGTGGGAAACATTAATTTAATTATATGGTATTATAGATATAGATAAGATGTGATATTTGATATATAAAACAAAAGAAAAATACAGAGAGTCAAAGAACATTGATAATAATTTATGTATAAAGAATCTATGTGTATTGTATTATAAAATAAATTATTAATTAGGTGGGGAGGGTAGGGTAGTGGTGGAGGTTTGTGTAGAAGATTGGTGGAGGGGTATGAGGTACTTAAATAATTTTGTGTAGTGTAGGTGGTATTTATTTTGGTGGAGAACGGCACAGGTGATAAAATTACGAGGGATATAAAGAAGCTTATAAAATTTTGTGAAAATTTGGAAGATTTTGGGGGTCAA

**>3394-21e.scf**

TACTGGGGGTTGGTGTAAAATAGTCAGGGTAGGAGATCTTGGTGGAGACATAAAATTATGGTAATAATTGTTTGGTGATATGGATAGTTTGGGGTGGAGTGATTGTGTAGGTGTGGTGTGGTGTGAGAGTTGGTGGGAGGGGTATTATGAGAGGGGTGTATTATAATATGGGTGGTGTATGTATTTTGTGGGAGGGATAGGGAGGGGTGTGGTGTAATAGGAGGGTGATGAAGGATTATTTAATTTATAGTTTTATATAGTTATAGATTATAGTTGTAGAAAGTTATATGAATATGCAACAACGAGAATGGCATTGTGAGTGTTAGAATAAAATTGAATTGTAAATATATTGTAGTGTTGTATATCATATAGATGAAATAATTATTTAGGGAGGGGTGGAGAGGTGTTATGATAGGAGGGGTGTGGGTGGAATTTAAGAAATAAAATTGGTGGGAGGATGTGGAGGATATAAAATTATTGGAGGGGTAGGAGAGGTTGGCAAAATTCACGAAAAA

**>3394-21j.scf**

TACTGGGGGTTGGTGTAAAATAGTCAGGGTGGGACCTCCGGGGAAATTTGGTAGGTGGTGAGTAGAAGGGTGTTGATTTTTGGTAGTATGTTGTGCGGGATGTATTAATATTCTTCTGGGGTGAGGGGTGTGGAAGAATTTTAATTTTATGATGTTTTAATTATTTTAGATATTTATGTGAATTAAATTATATGTAACAAATAACAGTGTGAGTTCTTGCAGTTGATTTTATAAGAACGTTCTAATGTATTATATTTATTTAGATAATATATATTAATGAGATATTTATAGATTGGTGGAGGGTGTTATTTCTTGTAGGAGGGGTAGATGAATTGTAGAGTTAAGATGGGAGGGGTAGAGGAGCAGCATGAAGGGATGTTGTAGTGGTGAGATTTAGATGGCGAAGATAAATTTATACAGTGTGAGAGGGGTAGATAGATATTTGGTGAAAATAAAATGTGTGGGGAAGATTAGTGGATAGGAGGAGGTAGATTTTATTAGATTTTATAAAATTTCGCCGAAAAATGACC

**>3394-21m.scf**

TACTGGGGGTTGGTGTAAAATAGTCAGGGTGGGATCTCCAAGATTTTGTGATAAAATGGTGGGAGGGCGGGAGAGAGATTAAATTTAAATTTATGATAAATAGTTAGAACATGGTGGTAGTGAGTTTATATAAAATCATAAAAACAAAGGACCGTAGTAGAAATAAAATGTGATCGTTCTTATAATTATATAGAGGGTAGTGTATCATATAATACTTAATTATTTTCCTTCAGGGGTGGGAGGGTGAAATTTATTTAGTGTATGGTAGATTATTATAGGGGTATATGTGATGCATTATATATAAAACAAAAACAAAAGAATCATCTAGAACGGGAGGTAATATAAGAAAATTATAAGATCTATCCTATAGGTAAAATATATATGTACCTTAAAATATTAATTTAGTAgGAGGGGTAGGGTAGTGgTGATGTTGTAGAGGGAGGGTATTTTATTTTTAAAATTGTTGGAGGGAGGTTGGGTGTAGATGGGTAGAAGATAAAAATCATAAAATTTGGGAGATTTTTGAACCCCTAA

**>3394-21r.scf**

GAGGTTGAGATTAATTATTTTAAGATATATGTTATTATATTATAGAACGTTCTTTGTAATATTTATATAAAATGCAACACGTGGTGATAGGTGGTATTGTAATATATAAAGAAATTTGAGATAAATAAAATCAATATACATATTATATATATTAAATGTTTATTATTTTGGTGGAGGGGTAGGAGGGGGAAAGCATTGATTT

**>3394-26h.scf**

TTAGTTTTATATACATTACATCACCACTACCCATTATAGTTTTAAACCATATATCAAATATTTATGTTCCACCCCCTTCATCATCCACCATCACACACCTCCCTCCTACCCTCCCAAATACTATTTTAATTTTATGACATCCACTAACACACTATTATTTACACAAATTTATTATCACATTTCTATATCAGTTTACACCCCCATTTGCGTTAATTATATTTTTATAAATTCATACACTATCACACCTAGATCATATTCTTAAATTAAATACCCACCCATCTTAACACCCCGCACCAAACCTTTATCATTTTATCTCCCAAAATCCTCGGGAATCCCACCCTGACTATTTTACACCA

**>3466-21g.scf**

GTTGGTGTAAAATAGTCAGGGTGGGATCTCCGGGAAAATGTGGAGGTAGGGTATGGTTGTAGAAAGATTGTTTTTAAACTTTATTTGTTTGGGGTGGGAGGGAAGCTAATTAATTTTATGGTTGAGGTATATTATATATTTAAAATAGAAGTGATAgAGGTGACATGTAATACAAAAAGATGATACAAAATAATAGATGTGAATTTTATGTAATCTATCCTATAGGGTGTAATATTATCTTAAAATATTATATATTAGAAACCAAGAGGGGTAGGAGGGGTGATGGAGTAGGGTAGGAGGGGTGGAAAAGATTTAGATATATAGTTATTAATATTATTGTTCTTATAAGATTTAATATAATATATACAACAGCGGTGATTTAGTGTAGATTAATTTGTGAAGTGATAGTTGTAAAGTTTGTGTAGTGATAAAGTATTATAATAAAATTGTATTTAGGGGGGAGGGGAACAGAGGGATAGGGGATAGAATTTATGAAAAATCACCCAAAAT

**>3466-21i.scf**

GTTGGTGTAAAATAGTCAGGGTGGGATCCCCGAGGATTTTGGGGAGATGATGGTGAATGTATGTGTGTTATGATTGTGTTGAAATTTATTTTGGGGAGGGTGGAGTTGGTTGTTAAATGGTGGTTGTATATAAAATTATTTGGGGTAGAAATATTAATGGTGGGTGGTGGTGGTGATGAGTTGGGTAGGGTTATTGGTGGGAGGGGTGGAGGGGTGATAGTTACGTGTGAAAGATTTGGTGTAGGAAATTATTTAATAATATGGTTTATTTAATGTTGTTAATTGATAATGTGTTGTATATATACATAAAACAAACAAAGTGGAATGTGAGCGATAATTATATAAATGTTCTTAATGTATCTATCCTATAGGTAATATCATAAATTTAATTTTGGTTATTTAATTATGGGAGGGGTAAATAATATTAGGGGTAGGAGGGATAGGAGGGATAGGAGGGATAAATGGTAGGAGCGAGAGGATAGGGGTATAAAATAATAAAAATTACCCAAAATTTGACCCA

**>3466-21j.scf**

TATGTTATTATATTATAGAACGTTCTTTGTAATATTTATATAAAATGCAACACGTGGTGATAGATGGTATTGTAATATATAAAGAAATTTGAGATAAATAAAATTAATATAGGTATTATAGATATTAAATGTTTATTATTTTTGGTGGAGGGGTAGGAGG

**>3466-21k.scf**

TACTGGGGGTTGGTGTAAAATAGTCAGGGTGGGATCCCCGAAGAATTAGGGTTGTTGTTGAAATTTAATTTTAATGAGGGTGGAAACGGTAAGTAGTTAAATTTTATTTTTGGATGGGTGGAGGAGGAGGGGTGTATATTATGGGAGGGGTGTAGATTGAGGGGTGTACAAAAATGGGGAGGGGTGATGAGGGACGGTGGTGGGTAGGAGGGTTGGGAGGGGTGTGTGGGAGTTATTTAATATTATGGTATTTTATAATTTATATATTTTATGAGTTAATTATATATTAGAATATAACACAGCGAGAGAACAATGTTAGAGATATATACAGAAAGTTCTTATATAATAAATTTAATCATGATGATATATAATAAATGAATATTATTTTAGCATGGGAGGGGTGACGGGTGGGAGGTAAGAGGGAGGGATGGTAGAAAGATATTTTATTTATTTTTGGAGGTGTTGTGTGGATGGGTGGAAAAGGTTTAGTGTGGGAGGGTGTAATTTGTAAAATTTCACGAAAAT

**>3466-21l.scf**

TACTGGGGGTTGGTGTAAAATAGTCAGGGTGGATCCCCGAGATTTTTGGGGGAGATGGAGATGGGTTGTATTTTAGGAATATTTATTTTATTTATTTGGATACTAGATAGGTTTATCATTTTATCTGTTAGTTCTGTTCATAGATTTGTTTTGTGTGTATAAATTTTGTAACATGGAAGGGATATATTTGGTATCATATAAATTAATTATTTTCTTGAGGGAGGGTGGAGGGTGGTGACGAAGAAATAATTTAGTAATATGGTAGGATATATTGTAAGAGTTAATGATAGAGGTTATAATATAATAAACACATAAAATCAGATAGACGAGATAATTAATATGTAATAAAAATAATAATATAAATTGTGTTATAATATTTTAATTAATTAATTTTAGGGGGAGGGTGTGGTAATAAGATAGGTGGAGGGGTGGGAGGGTAGGAGATGATTTAAATTTCTAAAAATATTTTGGGGTAGAGAGTTAGAATGGGTGGGAGGTGATGAATTTTAACAGAT

**>3466-21d.scf**

TACTGGGGGTTGGTGTAAAATAGTCAGGGTGGGATCCCCGAGAATTTTGTGAAATTTTGCGAGGTGGAAATTTATGGTGAGAAGATGATGGGTGGGTGTGGAGAGTTATTTGGTATATGGTATCTTATGTATTTTATTATTTAATGTGTAGAAATTAAAAACACAACTAGTGGTGTATGGTGGTACGAGAAGTAGATGATTATAATATTGTTCTTATGTGAATGTAGTGTATCTTAAATTATATTTGGTTTGGTTTGTAGGAGGGTAGGAGGGGTGCGGGGAGAATTAATAATATATGCTGTGATTATAAGATATATGGTTAGAACGATTATATAAAATAAAAAACACAGAACGAAACAAGGGAGAGAGATAAATAAAGTTCTTATTATAGTTCTTATGTATTATATAATATATTATAAAATTTATTAAATCTGTGGGAGGGGTAGGTGGGGTAGGTGGGGTAGGTGTAGAGGCGGTGTGTGATGGGGGATAGATTTATAAAATTTTATAAAAAATCACCAAAAT

**>3466-26d.scf**

TACTGGGGGTTGGTGTAAAATAGTCAGGGCGGGATCCCCAGGATTTTTGGGTAGAATGTGGAGGTGTGTTTGAAGAGGGAAATTTAGATATGGTATATTGTAATGTTTGTAAAATTGGGTTATGATATAGATGAACAAAATTATAACGCCGAGTAGAAAATAAATTATGTGATCGTTGTATTGTATTGGGTGAATTAGATGTAGGGTATCTTAAATAGTTGTTTTGTGTTGGTGTGTAGGAGGGGTTACGTGGGGTGGAGATAATTTATTATATAGTATGATATAGAGTTATATAGTTGTGATATTTATATTTTATACATAATAACAATGACGGGTAGAGATAGAAGAACAAAAATGTCATTATATAAAATTAGTGTATTGTGTATGATATTAATTTAATTATTTATTTAGACATGGGAGTGTGGAGGGGTAGGAGGGGTAGGAGATTTAATTTATGAAAATTGGTTGGGAGGGGTGGAGAATTAAGAGGGGCCAGAAGAATATTAAAATTTCGCCAAAAAA

**>3466-4.scf**

TACTGGGGGTTGGTGTAAAATAGTCAGGGTAGTATCTCCGAGAATTTTAGGAGTAGAGGGTGTATGAGGAGGAGAGGTGGGAGGGAGGGGTGAGGATAATTATTTTAATTATGATATTATAATAGATAGATACATATAGTTTTGATTATAAATATAACATGTGTAGAGTGCGGTAGATTAATTAGAGATAAGATAGAACGTTTATTGTTGGTATATTATGTTGTATGTTATAAAATTTAATTATTTGGTTTGGTGGGAGGAGTGGGTTGTGAGGGGTGGAGAAGAAAATATTATTTATATGGTTGTATAAGGATGTGATTTAATATTGGTGCATTATATATAACCTAAATCAAGAACGAGATGAACAGAAATTGATGAGTGAATTTTATGTAATATGTATAAATTGATATATATATTATAATTAAGAATTAATTTGGGGGAGGGTGGAGTAGGAGGAGTAGGATTAGAAGAGGGGTAGGGGTAGGAGGGGACAACACACAAAAATCACGAAAAATAGGACCCAATTTTAGTCGCAAAGTTCGATTTTGGGAGGGGCGTTCTGCGAA
