# Supplementary material for: Leishmania major and Trypanosoma lewisi infection in invasive and native rodents in Senegal
Source: PLoS Negl Trop Dis. 2018 Jun 29;12(6):e0006615. doi: 10.1371/journal.pntd.0006615 (PMC6042788; doi:10.1371/journal.pntd.0006615)
Supplement: S2 Text — All the sequences presented were obtained from R. rattus samples. (DOCX) [file pntd.0006615.s003.docx]

**Text S4. Sequences obtained by the mini-exon PCR amplification for *Trypanosoma lewisi* detection. All the sequences presented were obtained from *R. rattus* samples.**

>3193

CTTCGGACTTCGCTCTTCTTCTTTTTTTTTTTTTTTTCTGCCCCCCCCCCCCGGGGGGGG

CAAAACCCCTGCCCCCCCTTTTGGCCCACGCGGTGA

>3320

TTCGGACTTCGCTCTTCTTTTTTTTTTTATTTTTTTTCTGTACCCCCGCCCCCGGGCGGG

GGCCAAACCCCGGGCCCCCTTTTTGTTCCCCCTGCTGTCAAAAAA

>3432

TGGAAGGTCTTCGGACTTCGCTCTTCTTTTTTTTTTTATTTTTTTTTTGACCCCCCCCCC

CGGGGGGGGGCAAAACCCTGCCCCCCCTTATTGCCCCCGCTGTAGACGAAA

>3433

TTCGGACTTCGCTCTTCTTTTTTTTTTTATTTTTTTTTTGCCCCCCCCCCCCGGGGGGGG

GCAAAACCCTGCCCCCCCTTATTGCCCCCGCTGTCAAAAA

>3470

CGGACTTCGCTCTTCTTTTTTTTCATTTTTTTCTGTACCTCCGTCCCCGGTCGGGGGCCA

AACCCTGGCCCCCCTTATTGCTCACGCTGCTGTC
