# Supplementary figures and images for: Leishmania major and Trypanosoma lewisi infection in invasive and native rodents in Senegal
Source: PLoS Negl Trop Dis. 2018 Jun 29;12(6):e0006615. doi: 10.1371/journal.pntd.0006615 (PMC6042788; doi:10.1371/journal.pntd.0006615)

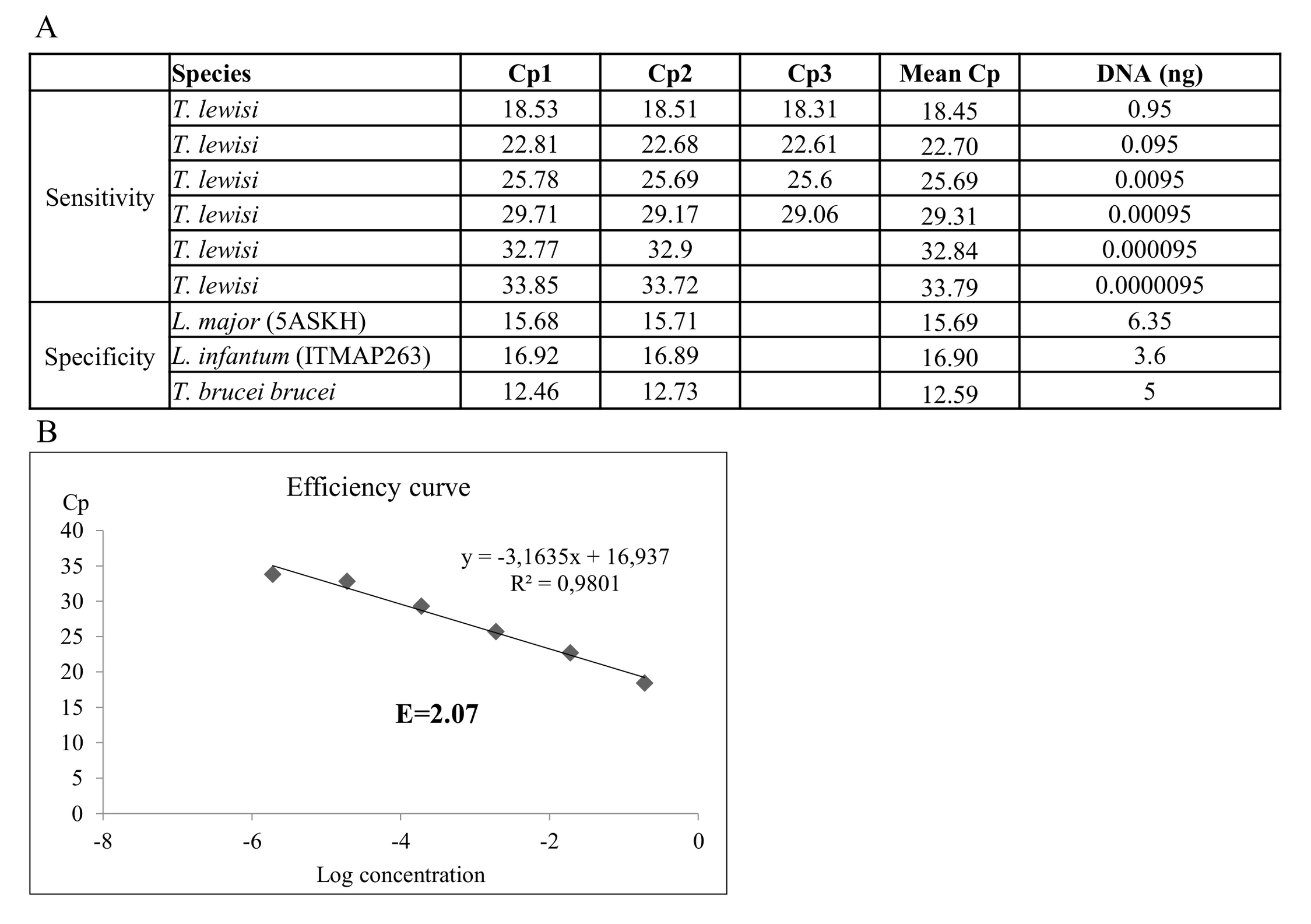

Supplement: S1 Fig — (A) Cp values of amplification of a T. lewisi DNA scale and of other trypanosomatids. (B) Efficiency curve. This method was chosen for its robustness and sensitivity (<9.5fg/μl). However, this PCR was not specific for T. lewisi, but detected also other trypanosomes as well as Leishmania. (TIF) [file pntd.0006615.s004.tif]

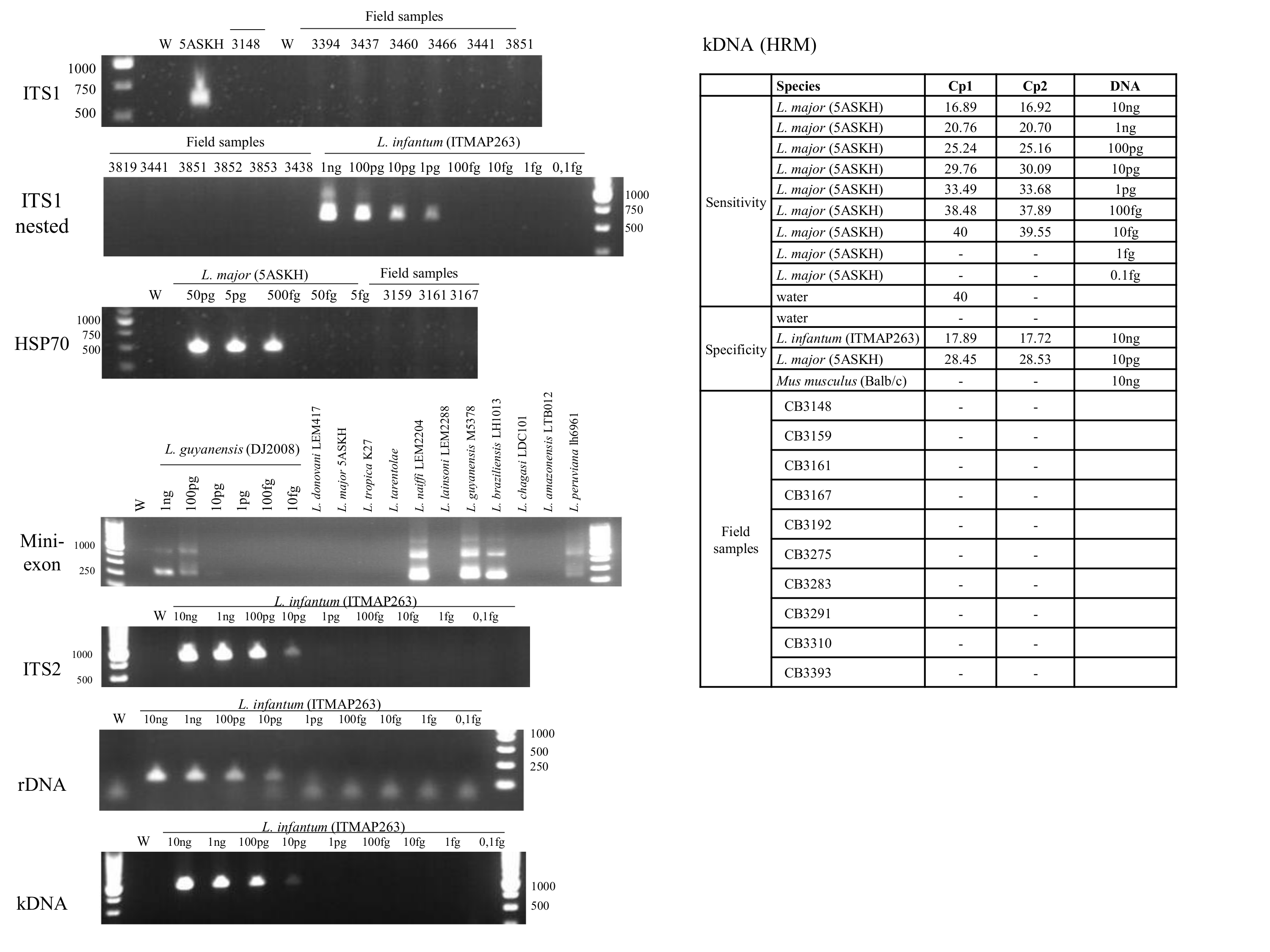

Supplement: S2 Fig — Field samples were chosen among samples that were positive with the nested PCR used for the Leishmania diagnosis and tested with other molecular methods. The sensitivity of these protocols was not sufficient to confirm the diagnosis made with the nested PCR on kDNA minicircles.W stands for water (negative control). Primers were the following: ITS1: LITSR, ITS1R [109]; nested ITS1 step 1: LITSR, LITSV, step 2: LITSR, L5.8S [110]; HSP70: forward [111], HSP70ant [112]; mini-exon: Fme, Rme [113]; ITS2: LGITSF2, LGITSR2 [114]; rDNA: rDNA-10F, rDNA-14R [115]; kDNA: L.MC-1S, L.MC-1R [116]; kDNA (real-time PCR HRM): MLF, MLR [117]. (TIF) [file pntd.0006615.s005.tif]

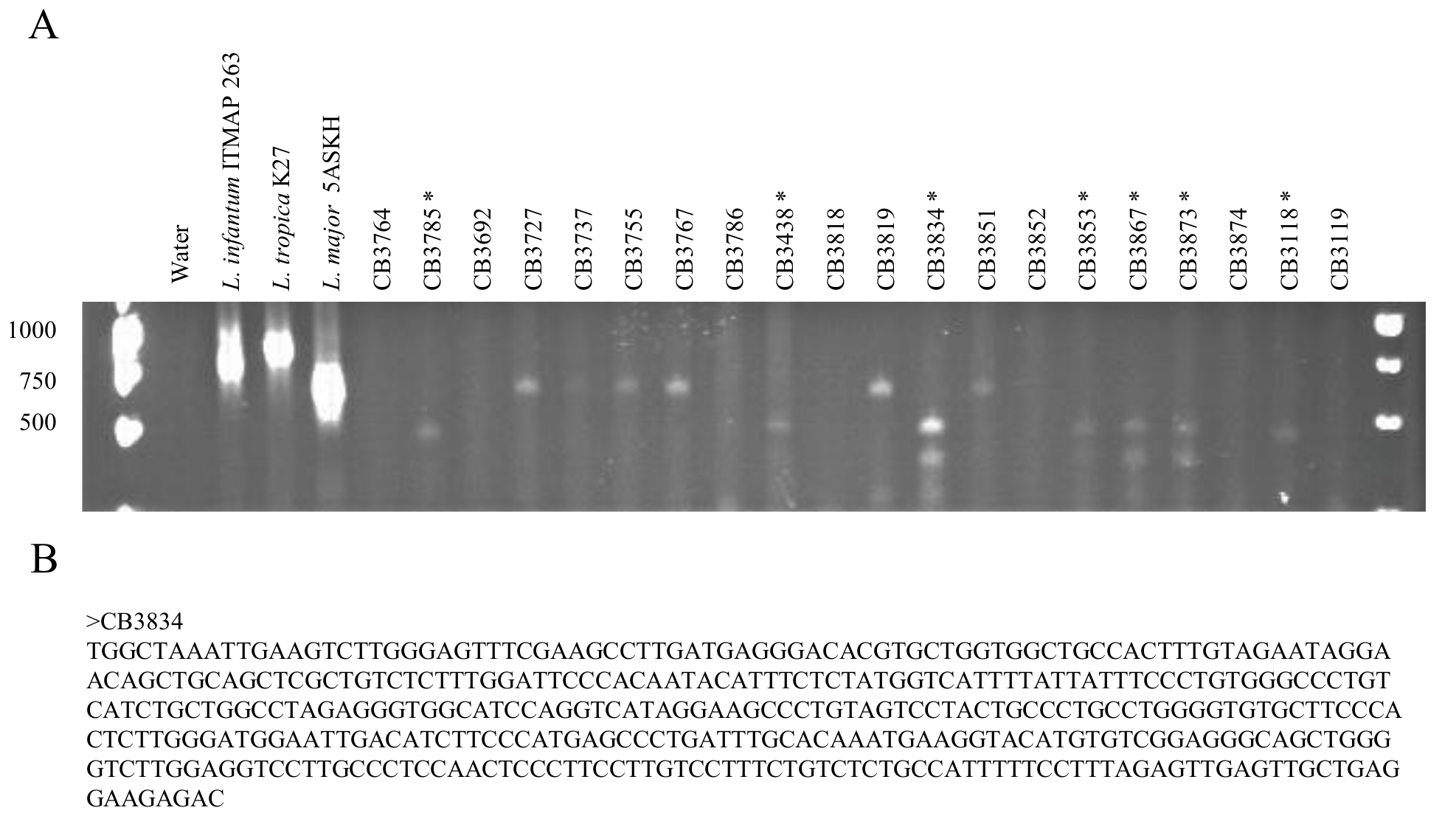

Supplement: S3 Fig — (A) Exampe of electrophoresis gel showing unspecific amplification obtained from a few field samples (7%) with the nested PCR on kDNA minicircles (samples marked with *, lower bands). (B) Example of sequence obtained from the sample CB3834 and presenting 85% homology on 406b with mouse DNA sequences found in public databases (for example GenBank AL772311.19). Overall, seven amplification products from seven field samples were directly sequenced and showed homology with rodent DNA. (TIF) [file pntd.0006615.s006.tif]

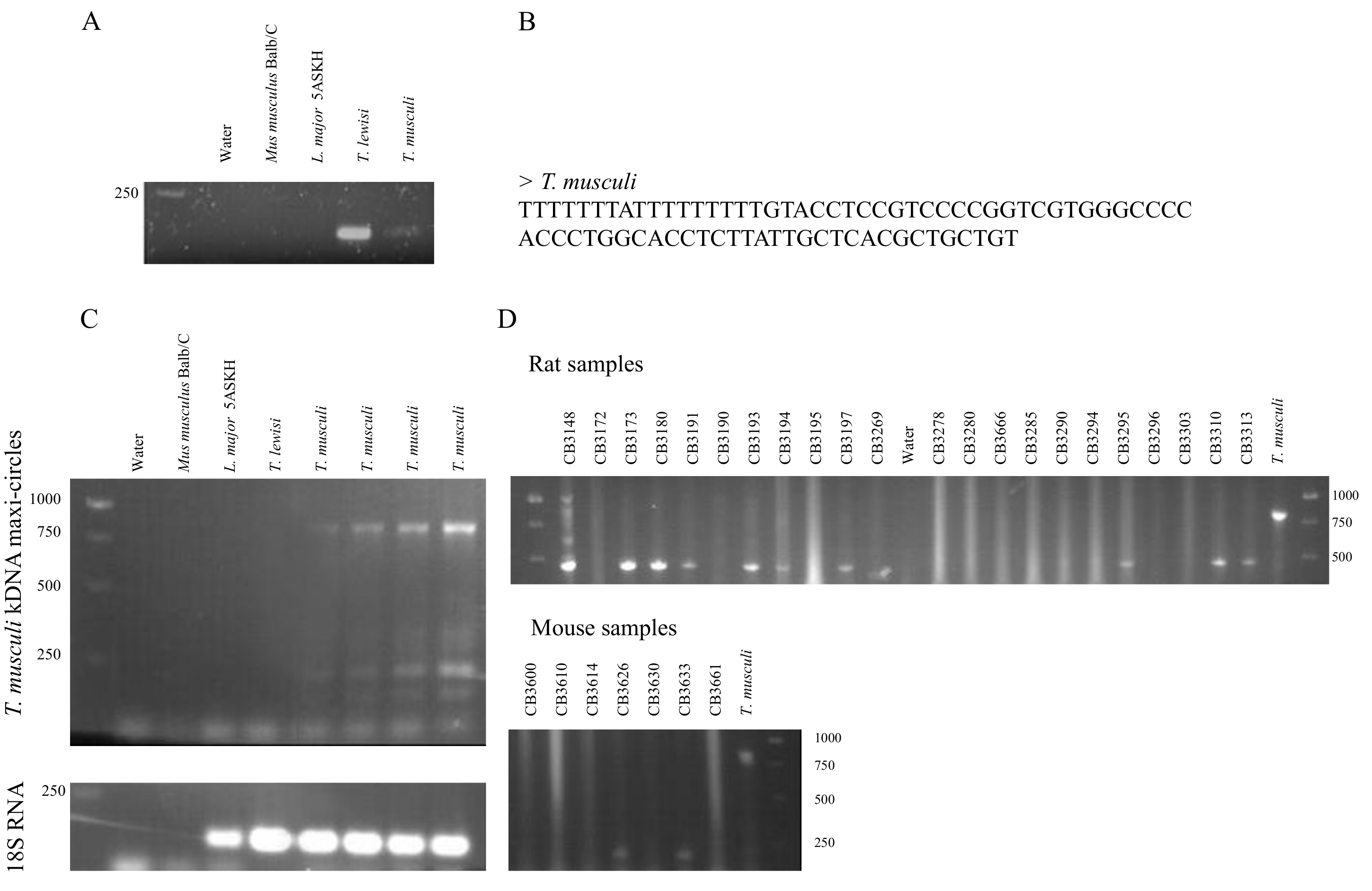

Supplement: S4 Fig — (A) Electrophoresis gel of the T. lewisi mini-exon PCR performed on T. musculi and other reference strains. Our T. lewisi diagnosis was not strictly specific and could amplify T. musculi. (B) Sequencing of the mini-exon PCR product from T. musculi. There were too many variability and too few data in databases to differentiate T. lewisi and T. musculi on the basis of mini-exon sequencing. (C) Electrophoresis gel of amplification of different reference strains with T. musculi kDNA maxi-circles PCR (upper part). The four T. musculi samples were extracted independently from the same blood sample of an infected mouse. Primers used were TM1F, TM1R [96]. In the lower part, amplification of the same samples with the 18S rRNA PCR showed that the T. musculi kDNA maxi-circles PCR seemed to be not very sensitive. (D) Electrophoresis gel of T. musculi kDNA maxi-circles performed on field samples (upper part: rat samples; lower part: mouse samples). This PCR gave frequent (33/66, 50%) non specific amplification, probably from rodents DNA. We obtained no band of size corresponding to specific amplification. (TIF) [file pntd.0006615.s007.tif]
