# Supplementary material for: Leishmania major and Trypanosoma lewisi infection in invasive and native rodents in Senegal
Source: PLoS Negl Trop Dis. 2018 Jun 29;12(6):e0006615. doi: 10.1371/journal.pntd.0006615 (PMC6042788; doi:10.1371/journal.pntd.0006615)
Supplement: S1 Table — For Leishmania diagnosis, the nested PCR method to amplify the minicircle kinetoplastic DNA (kDNA) variable region described by Noyes et al. [51] was used, and then amplicons were separated by electrophoresis before cloning. * BLAST results were retained only if they corresponded to the sequences of reference strains or to sequences of isolates described in peer-reviewed articles and well characterized in terms of species, origin and hosts, in order to avoid misidentifications. ** When the results obtained with one database were highly significant and with high score whereas low similarity was found with the second one, only the highly significant results are provided. (DOCX) [file pntd.0006615.s001.docx]

**Table S1. Blast analysis of the sequences obtained by PCR amplification for *Leishmania* detection.**

* BLAST results were retained only if they corresponded to the sequences of reference strains or to sequences of isolates described in peer-reviewed articles and well-characterized in terms of species, origin and hosts, in order to avoid misidentifications

** When the results obtained with one database were highly significant and with high score whereas low similarity was found with the second one, only the highly significant results are provided.
